# Genome-wide association study for cashmere traits in Inner Mongolia cashmere goat population reveals new candidate genes and haplotypes

**DOI:** 10.1186/s12864-024-10543-4

**Published:** 2024-07-02

**Authors:** Youjun Rong, Xinle Wang, Qin Na, Xiaofang Ao, Qincheng Xia, Furong Guo, Mingxuan Han, Rong Ma, Fangzheng Shang, Yan Liu, Qi Lv, Zhiying Wang, Rui Su, Yanjun Zhang, Ruijun Wang

**Affiliations:** 1 College of Animal Science, Inner Mongolia Agricultural University, Hohhot, 010018 China; 2 Key Laboratory of Mutton Sheep Genetics and Breeding, Ministry of Agriculture, Hohhot, 010018 China; 3 Key Laboratory of Goat and Sheep Genetics, Breeding and Reproduction in Inner Mongolia Autonomous Region, Hohhot, 010018 China; 4 Northern Agriculture and Livestock Husbandry Technology Innovation Center, Hohhot, 010018 China; 5 Inner Mongolia Autonomous Region Agricultural and Animal Husbandry Technology Extension Center, Hohhot, 010018 China; 6 College of Vocational and Technical, Inner Mongolia Agricultural University, Baotou, 014109 China

**Keywords:** Inner Mongolia cashmere goats, Cashmere traits, GWAS, Potential candidate genes, Haplotype block, KASP

## Abstract

**Background:**

The cashmere goat industry is one of the main pillars of animal husbandry in Inner Mongolia Autonomous Region, and plays an irreplaceable role in local economic development. With the change in feeding methods and environment, the cashmere produced by Inner Mongolia cashmere goats shows a tendency of coarser, and the cashmere yield can not meet the consumption demand of people. However, the genetic basis behind these changes is not fully understood. We measured cashmere traits, including cashmere yield (CY), cashmere diameter (CD), cashmere thickness (CT), and fleece length (FL) traits for four consecutive years, and utilized Genome-wide association study of four cashmere traits in Inner Mongolia cashmere goats was carried out using new genomics tools to infer genomic regions and functional loci associated with cashmere traits and to construct haplotypes that significantly affect cashmere traits.

**Results:**

We estimated the genetic parameters of cashmere traits in Inner Mongolia cashmere goats. The heritability of cashmere yield, cashmere diameter, and fleece length traits of Inner Mongolia cashmere goats were 0.229, 0.359, and 0.250, which belonged to the medium heritability traits (0.2 ~ 0.4). The cashmere thickness trait has a low heritability of 0.053. We detected 151 genome-wide significantly associated SNPs with four cashmere traits on different chromosomes, which were very close to the chromosomes of 392 genes (located within the gene or within ± 500 kb). *Notch3*, *BMPR1B*, and *CCNA2* have direct functional associations with fibroblasts and follicle stem cells, which play important roles in hair follicle growth and development. Based on GO functional annotation and KEGG enrichment analysis, potential candidate genes were associated with pathways of hair follicle genesis and development (Notch, P13K-Akt, TGF-beta, Cell cycle, Wnt, MAPK). We calculated the effective allele number of the Inner Mongolia cashmere goat population to be 1.109–1.998, the dominant genotypes of most SNPs were wild-type, the polymorphic information content of 57 SNPs were low polymorphism (0 < PIC < 0.25), and the polymorphic information content of 79 SNPs were moderate polymorphism (0.25 < PIC < 0.50). We analyzed the association of SNPs with phenotypes and found that the homozygous mutant type of SNP1 and SNP3 was associated with the highest cashmere yield, the heterozygous mutant type of SNP30 was associated with the lowest cashmere thickness, the wild type of SNP76, SNP77, SNP78, SNP80, and SNP81 was associated with the highest cashmere thickness, and the wild type type of SNP137 was associated with the highest fleece length. 21 haplotype blocks and 68 haplotype combinations were constructed. Haplotypes A2A2, B2B2, C2C2, and D4D4 were associated with increased cashmere yield, haplotypes E2E2, F1F1, G5G5, and G1G5 were associated with decreased cashmere fineness, haplotypes H2H2 was associated with increased cashmere thickness, haplotypes I1I1, I1I2, J1J4, L5L3, N3N2, N3N3, O2O1, P2P2, and Q3Q3 were associated with increased cashmere length. We verified the polymorphism of 8 SNPs by KASP, and found that chr7_g.102631194A > G, chr10_g.82715068 T > C, chr1_g.124483769C > T, chr24_g.12811352C > T, chr6_g.114111249A > G, and chr6_g.115606026 T > C were significantly genotyped in verified populations (*P* < 0.05).

**Conclusions:**

In conclusion, the genetic effect of single SNP on phenotypes is small, and SNPs are more inclined to be inherited as a whole. By constructing haplotypes from SNPs that are significantly associated with cashmere traits, it will help to reveal the complex and potential causal variations in cashmere traits of Inner Mongolia cashmere goats. This will be a valuable resource for genomics and breeding of the cashmere goat.

**Supplementary Information:**

The online version contains supplementary material available at 10.1186/s12864-024-10543-4.

## Introduction

Inner Mongolia cashmere goat is an excellent local livestock breed after long-term natural selection and artificial systematic breeding. It was included in the "National List of Livestock and Poultry Breeds Protection" in 2000 and the "National List of Livestock and Poultry Genetic Resources Protection" in 2006. In 2008, the breeding area of the Inner Mongolia Cashmere Goat was designated as a national breeding herd and protected area. Inner Mongolia cashmere goat is the state expressly prohibited the export of livestock species [1]. The cashmere produced by Inner Mongolia cashmere goats has the advantages of slimness, softness and good luster, which is loved by domestic and foreign consumers. It is the only export livestock product with pricing rights in China, which plays an important role in economic development. With the current transformation and upgrading of the livestock industry and the improvement of people's consumption demand, the current cashmere production capacity of Inner Mongolia cashmere goats can not meet the current demand. Therefore, to improve the performance of cashmere production has become one of the problems to be solved, and the genetic improvement of cashmere production performance has become the focus of breeding work.

GWAS, as an important tool for genetic localization, has been playing an important role in resolving the genetic basis of economically important traits in livestock and poultry. Cashmere trait is an important indicator of fiber yield and quality during goats domestication. However, the gene regulation behind the selection of cashmere traits is still unclear. Wang et al. [2] used the Illumina Goat 52 K SNP chip to detect 192 Inner Mongolia cashmere goats. Through GWAS, they found that four SNPs were significantly associated with cashmere fiber length, fiber diameter, and yield. These SNPs were located in genes such as FGF12, SEMA3D, EVPL, and SOX5, which may have potential effects on the cashmere traits of Inner Mongolia cashmere goats. At present, although there are a few studies on cashmere traits, it is still necessary to expand the population in different regions, increase the sequencing depth, and use statistical methods to mine the genetic markers that actually influence the phenotype of cashmere goats. In this study, 404 Inner Mongolia cashmere goat individuals were subjected to whole genome resequencing, and the obtained SNPs were quality-controlled and then the linear mixed model of GCTA software was used to analyze the association of cashmere traits respectively, in order to search for the causal variation and potential candidate genes of the cashmere traits, which provided some theoretical foundation and basis for the subsequent breeding work and genetic study of Inner Mongolia cashmere goats.

## Methods

### Source of experimental animal and phenotype data

**Fig. 1:**
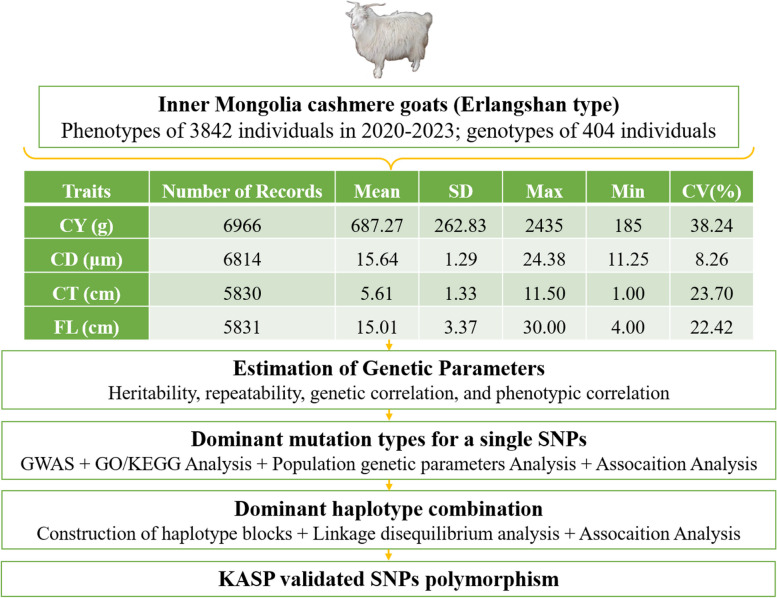
Descriptive statistics for phenotypic values of cashmere traits in Inner Mongolia cashmere goats population and analysis pipeline. Diagram of the pipeline used to identify single SNPs and haplotype combinations significantly associated with cashmere traits. CY(cashmere yield), CD(cashmere diameter), CT(cashmere thickness), FL(fleece length)

The experimental goats in this study were all from Erlangshan Ranch of Inner Mongolia Beiping Textile Co., Ltd. (Inner Mongolia cashmere goat Erlangshan type national protected breeding farm), and the breeds of cashmere goats raised were all Erlangshan type Inner Mongolia cashmere goats. We screened 404 Inner Mongolia cashmere goats for resequencing from 9 herds at Erlangshan Ranch. Of these, 84 were rams and 320 were ewes. The ram population consists of 75 one-year-old rams, 4 two-year-old rams, and 5 three-year-old rams; The ewe population consists of 92 ewes aged one year, 54 ewes aged two years, and 174 ewes aged three years. The number of cashmere goats in herd 1–9 was 9, 75, 41, 49, 14, 29, 46, 92, 49. According to the method described in Table 1, the phenotypic data of cashmere traits of 3842 individuals from 2020 to 2023 were determined, including 1401 males and 2441 females. The genealogy can be traced back to two generations. Regarding phenotypes, there were 6966 records for CY, 6814 records for CD, 5830 records for CT, and 5831 records for FL, respectively. Descriptive statistical analysis of the phenotype and analysis pipeline is presented in Fig. 1. We selected 96 individuals of Inner Mongolia cashmere goats with complete phenotypic records from the 10th herd to extract DNA. After passing the quality test, DNA was placed in dry ice and sent to Beijing Compsen Biotechnology Co., Ltd. for KASP genotyping.

**Table 1:** Description of the four cashmere traits recorded on Inner Mongolia cashmere goats

Cashmere traits	Description	Measuring device
Cashmere yield	The weight of the cashmere measured after the cashmere is cleaned	Electronic scales
Cashmere diameter	The average diameter of a goat's cashmere	Cashmere fineness analyzer
Cashmere thickness	The average distance between the bottom and top of the cashmere	Measuring stick
Fleece length	The average linear distance from the root to the tip of fleece	Measuring stick

### Estimation of genetic parameters

REML algorithm of ASReml software and animal model were used to estimate the genetic parameters of each trait. The model is as follows:

$$\mathrm y=\;\mathrm{Xb}\;+\;{\mathrm Z}_1\mathrm a\;+\;{\mathrm Z}_2\mathrm p\;+\;\mathrm e$$

Where y is a vector of phenotypic values; b is a vector of fixed effects (including herd-year of birth and age); a is the vector of additive genetic effects, and its normal distribution is a ~ N(0, $\text{A}\sigma_\text{a}^2$), ${\upsigma }_{\text{a}}^{2}$ is the additive genetic variance of traits, and A is the additive genetic correlation matrix between individuals. P is a vector of the permanent environmental effect, its normal distribution is p ~ N(0, $\text{I}\sigma_\text{P}^2$), ${\upsigma }_{\text{p}}^{2}$ is the permanent environmental variance, I is the identity matrix; X, Z1, and Z2 are the correlation matrices of b, a, and p, respectively; e is the residual vector, its normal distribution is e ~ N(0, $\text{I}\sigma_\text{e}^2$), I is the identity matrix, ${\upsigma }_{\text{e}}^{2}$ is the residual variance.

The estimated variance components are calculated using the following formula to calculate the corresponding genetic parameters:

$$\text{Heritability}:{h}^{2}=\frac{{\sigma }_{a}^{2}}{{\sigma }_{a}^{2}+{\sigma }_{e}^{2}}$$

$$\text{Repeatability}:{r}_{e}=\frac{{\sigma }_{a}^{2}+{\sigma }_{ep}^{2}}{{\sigma }_{a}^{2}+{\sigma }_{ep}^{2}+{\sigma }_{et}^{2}}$$

$$\text{Genetic correlation}:{r}_{A}=\frac{Co\upsilon ({a}_{1},{a}_{2})}{\sqrt{{\sigma }_{{a}_{1}}^{2}{\sigma }_{{a}_{2}}^{2}}}$$

$$\text{Phenotypic correlation}:{r}_{P}=\frac{Co\upsilon ({p}_{1},{p}_{2})}{\sqrt{{\sigma }_{{p}_{1}}^{2}{\sigma }_{{p}_{2}}^{2}}}$$

Among them, *h*^*2*^ represents heritability; ${\sigma }_{a}^{2}$ represents additive genetic variance; ${\sigma }_{e}^{2}$ represents the residual variance; ${r}_{e}$ represents repeatability; ${\sigma }_{ep}^{2}$ represents the permanent environmental variance; ${\sigma }_{et}^{2}$ represents temporary environmental variance; ${r}_{A}$ represents genetic correlation between traits. *Cov(a*_*1*_*, a*_*2*_*)* represents the additive effect covariance of traits *a*_*1*_ and *a*_*2*_. ${\sigma }_{{a}_{1}}^{2}$ represents the additive variance of trait *a*_*1*_. ${\sigma }_{{a}_{2}}^{2}$ represents the additive variance of trait *a*_*2*_. ${r}_{P}$ represents the phenotypic correlation between traits. *Cov(p*_*1*_*, p*_*2*_*)* represents the phenotypic covariance of *p*_*1*_ and *p*_*2*_. ${\sigma }_{{p}_{1}}^{2}$ represents the phenotypic variance of the trait *p*_*1*_. ${\sigma }_{{p}_{2}}^{2}$ represents the phenotypic variance of the trait *p*_*2*_.

### Genomic DNA extraction and quality control

Ear tissue samples were collected from 404 individuals of Inner Mongolia cashmere goats (Erwangshan type). During the sampling, the fleece of Inner Mongolia cashmere goat's ear posterior margin was cut off first, disinfected with 75% ethanol, and 0.5 g of ear tissue near 1/3 of the ear tip was cut off with ear amputer and placed into 1.5 mL cryotube. Because of the small distribution of blood vessels at the back edge of the ear, there will be no bleeding after sampling. After sampling, apply pain medication to the wound immediately, and the animals were released and not sacrificed in the process. All experiments and procedures were carried out following the Scientific Research and Academic Ethics Committee of Inner Mongolia Agricultural University and the Biomedical Research Ethics of Inner Mongolia Agricultural University (Approval No. [2020] 056). No specific permissions were required for these activities, and no endangered or protected species were involved. All samples were stored in liquid nitrogen immediately after collection, and transported to the laboratory for long-term storage at -80℃. DNA was extracted from the ear tissue samples using the phenol–chloroform method, and DNA concentration, the absorption wavelength ratios of the highest absorption peaks of nucleic acids, proteins, carbohydrates (260 nm/230 nm) and phenolics (260 nm/280 nm) were detected by a spectrophotometer (NanoDrop2000).The 1% agarose gel was used to detect and evaluate the DNA quality, Store qualified DNA samples in a -20 ℃ refrigerator for future use.

### Library construction and on-line sequencing

After the qualified genomic DNA samples were processed, the genomic DNA was randomly interrupted into 350 bp length fragments by Covaris ultrasonic crusher. The DNA fragments were subjected to end repair, addition of poly(A), addition of sequencing adapters, purification, PCR amplification and other steps to complete the whole library preparation. After the library was constructed, Qubit 2.0 was used for preliminary quantification, and qPCR was used to accurately quantify the effective concentration of the library to ensure the quality of the library. After the quality of the library passed the test, sequencing was carried out using the DNBSEQ-T7 sequencing platform, and the sequencing mode was PE150 mode.

### Identification, screening and annotation of mutation sites

Filter Raw reads data into Clean reads data (-n 10 -q 20 -u 40) using fastp software (V0.20.0) [3], establish a genome index for the reference genome, and the Burrows-Wheeler Aligner (BWA) software (V0.7.17) [4] was used to compare (mem -T 20 -5a) the quality controlled Clean reads with the goat reference genome (ARS1, GCF_001704415.1); using SAMtools software (V1.8–20) [5] to convert (view -b) the compared sam files into bam files and sort (sort) the bam files; MarkDuplicates program in Genome Analysis Toolkit (GATK) software (V3.8) [6] was used to remove duplicates (-MAX_FILE_HANDLES 500 –VALIDATION_STRINGENCY SILENT –REMOVE_DUPLICATES) from the sorted bam file to obtain the final bam file; the final bam file was indexed, and SNP variant detection was carried out by using the HaplotypeCaller module (–emit-ref-confidence GVCF) in the GATK software. Convert GVCF files to VCF files using the GenotypeGVCFs module (-new-qual True) in the GATK software. Then filtered (–filter-expression "DP < 8.0 || DP > 200.0 || QD < 2.0 || MQRankSum < -12.5 || ReadPosRankSum < -8.0 || MQ < 40.0 || FS > 60.0 || SOR > 3.0" –filter-name "Filter") by using the VariantFiltration module after obtaining the VCF file. Functional annotation of the detected gene variants was performed using the ANNOVAR software package [7]. Based on the position of the variant site on the reference genome and the gene position information on the reference genome, the region of the genome in which the variant site occurs (intergenic region, intronic region, or CDS region, etc.), as well as the effect of the variant (synonymous non-synonymous mutation, etc.) can be obtained.

### Data quality control, genetic relationship analysis based on IBS distance matrix and G matrix, PCA analysis

The obtained genotyping data were quality controlled using Plink (V1.90) software [8] to exclude individuals with genotype detection rate (call rate) < 98%, SNPs with call rate < 98%, SNPs with minimum allele frequency (MAF) < 5%, and SNPs with *P*-value of the Hardy Weinberg Equilibrium (HWE) test < 10^–6^. The identity by state (IBS) matrix and the G matrix of genomic relatedness were constructed by using Plink (V1.90) software. Finally, R (V3.6.0) [9] plotting was utilized for the visual presentation of genetic distance and kinship results. The "–pca 3" parameter of Plink (V1.90) software was used to calculate the first three principal components, and the PCA plot was drawn using R (V3.6.0).

### Genome-wide association study

The estimated breeding values (EBV) for CY, CD, CT, and FL traits were estimated according to average information restricted maximum likelihood (AI-REML) using the single traits repeatability model and the DMUAI module of DMU software [10], The corrected phenotypic value is obtained by adding the estimated breeding value and the residual, as described below:

$${\text{y}}_{\text{c}} =\text{ Xb }+ {\text{Z}}_{1}\text{a }+ {\text{Z}}_{2}\text{p }+\text{ e}$$

where y_c_ is the vector of corrected phenotypic value; b is the vector of fixed effects, including herd-year of birth and age; a is the vector of additive genetic effect with a normal distribution is a ~ N(0, $\text{A}\sigma_\text{a}^2$), ${\upsigma }_{\text{a}}^{2}$ is the additive genetic variance of the trait, and A is the additive genetic relationship matrix between individuals; p is the vector of permanent environmental effect with a normal distribution is p ~ N(0, $\text{I}\sigma_\text{P}^2$), ${\upsigma }_{\text{p}}^{2}$ is the variance of permanent environment, I is the unit matrix; X, Z_1_ and Z_2_ are the incidence matrices of b, a and p, respectively; e is the residual vector with a normal distribution is e ~ N(0, $\text{I}\sigma_\text{e}^2$), I is the unit matrix, and ${\upsigma }_{\text{e}}^{2}$ is the residual variance.

Association analysis between SNPs and individual traits (CY, CD, CT, and FL) was performed using the fastGWA-mlm model in the GCTA (V1.94.0beta) software [11].

$$\text{y}={\text{X}}_{\text{snp}}{\upbeta }_{\text{snp}}+\text{g}+\text{e}$$

Where y is an n × 1 vector of mean-centered phenotypes; X_snp_ is a vector of mean-centered genotype variables of a variant of interest with its effect β_snp_; g is a vector of the total genetic effects captured by SNP-derived genetic relationship matrix (GRM) with g ~ N(0, $\pi\sigma_{\text{g}}^{2}$); π is a SNP-derived GRM vector of with all of the small off-diagonal elements set to 0; e is a vector of residuals with e ~ N(0, $\text{I}\sigma_\text{e}^2$).

The threshold of significance for determining GWAS is too stringent due to the use of the Bonferroni correction method. In this study, the threshold for genome-wide significant associations was adjusted to *P* = 1 × 10^–6^. The genome inflation factor (i.e., λ) for the test statistic was calculated from the slope of the linear regression between the observed and theoretical quartiles in R (V4.2.2). Significant SNPs are shown as threshold lines in the Manhattan plot. Manhattan plots and quantile–quantile (QQ) plots were generated by the CMplot package in R (https://github.com/YinLiLin/R-CMplot, 20 January 2019).

### Kompetitive allele specific PCR

A novel competitive SNP genotyping method (KASP) was used to verify 8 SNPs that were significantly associated with the cashmere yield, cashmere diameter, cashmere thickness, and fleece length of Inner Mongolia cashmere goats.

All DNA samples diluted to a similar concentration equivalent to 40 ng/sample were utilized for genotyping. Primer5 software was used to design SNP-specific targeted KASP detection primers, and the primers sequence was shown in Table 2.

**Table 2:** Selected SNPs used in the study for verification of the Inner Mongolia cashmere goats

Locus	Primer	Primer sequence
chr7_g.102631194A > G	Primer_AlleleFAM	GAAGGTGACCAAGTTCATGCTCTTCATGGTGGGTAGGTGACAGTA
	Primer_AlleleVIC	GAAGGTCGGAGTCAACGGATTCTTCATGGTGGGTAGGTGACAGTG
	Primer_Common	GGTTCATCCTTTTTGTCGCATAGT
chr10_g.82715068 T > C	Primer_AlleleFAM	GAAGGTGACCAAGTTCATGCTGGAGGGAAGAGGGGGCCT
	Primer_AlleleVIC	GAAGGTCGGAGTCAACGGATTGGAGGGAAGAGGGGGCCC
	Primer_Common	CCTCTGCTTTCTCTCAAGAGCTAA
chr1_g.124483769C > T	Primer_AlleleFAM	GAAGGTGACCAAGTTCATGCTCATTATTATTTCTCCTCAGCATCATTATATC
	Primer_AlleleVIC	GAAGGTCGGAGTCAACGGATTCATTATTATTTCTCCTCAGCATCATTATATT
	Primer_Common	CGAAACAGAAACTAGCAGTCACTC
chr6_g.30463541A > T	Primer_AlleleFAM	GAAGGTGACCAAGTTCATGCTGGCTGTCTCCTCTCCCAAGT
	Primer_AlleleVIC	GAAGGTCGGAGTCAACGGATTGGCTGTCTCCTCTCCCAAGA
	Primer_Common	GCACAGGATGGTTCTGTAATGATG
chr24_g.12811352C > T	Primer_AlleleFAM	GAAGGTGACCAAGTTCATGCTAAGATGTATAGGTTCAACCCCAGG
	Primer_AlleleVIC	GAAGGTCGGAGTCAACGGATTAAGATGTATAGGTTCAACCCCAGA
	Primer_Common	TCCAGACAAAGATACTGGAATGGG
chr24_g.14180758C > T	Primer_AlleleFAM	GAAGGTGACCAAGTTCATGCTAAACCAAGGAAATTCCAGAAAGAC
	Primer_AlleleVIC	GAAGGTCGGAGTCAACGGATTAAACCAAGGAAATTCCAGAAAGAT
	Primer_Common	TGATCCACACAAAGGCTATAGTGT
chr6_g.114111249A > G	Primer_AlleleFAM	GAAGGTGACCAAGTTCATGCTCCTCTCATTGAGATGGGTTGCA
	Primer_AlleleVIC	GAAGGTCGGAGTCAACGGATTCCTCTCATTGAGATGGGTTGCG
	Primer_Common	TGGTCAACACTCCAGAAGAAGATT
chr6_g.115606026 T > C	Primer_AlleleFAM	GAAGGTGACCAAGTTCATGCTCTCTGGACCCCATCTGGATGT
	Primer_AlleleVIC	GAAGGTCGGAGTCAACGGATTCTCTGGACCCCATCTGGATGC
	Primer_Common	CCTCAGCTGACGTTTGGTGA

The RT-PCR amplification was performed using KASP Master Mix (AQP-001L, HiGeno 2 × Probe Mix, JasonGen, CN) with 0.1 μM of each primer pair and 40 ng of DNA template per reaction. Each RT-PCR reaction volume of 5 μL consisted of 2.5 μL of the KASP Master Mix, 0.07 μL primer Mix, and 2.5μL DNA template, which was processed under the following amplification conditions: holding (94 ℃/15 min, 1 cycle), touchdown cycles (94 ℃/20 s: touchdown55-61 °C, -0.6 ℃ per cycle/60 s, 10 cycles). Also, there was further cycling (94 ℃/20 s and 55 ℃/60 s, 26 cycles), followed by a post read stage at (35 °C/60 s). The amplification was carried out using QuantStudio™ 7 Flext Real-Time PCR System (Applied Biosystems, Thermo Fisher Scientific™, USA). The endpoint fluorescence results were analyzed with QuantStudio software V1.7.2 (https://downloads.thermofisher.cn/QuantStudio_Real_Time_PCR_Software/v1.7.2/QuantStudio_C02R31_10082021.0257.zip).

### Statistical analyses

To identify potential candidate genes associated with cashmere traits, significantly associated SNPs were annotated using Bedtools (V2.30.0) software [12] based on the goat ARS1 version of the genome. SNPs were localized to the identified genes, and only those genes that were located within a 1 Mb (± 500 kb) range around each SNP were considered. In the case of more than two identified genes within the identified range, preference was given to the gene where the SNP was located, or the gene where the SNP was closest. However, if the SNP was located between two genes, both genes were selected and the remaining gene was discarded.

In addition, candidate genes were analyzed for GO function annotation and KEGG enrichment using the clusterProfiler package [13] in R (V4.2.2). KEGG pathways and GO biological processes of candidate genes were manually searched to infer potential gene functions. Based on the principles of calculating parameters such as allele frequency, genotype frequency, homozygosity (Ho) and heterozygosity (He), we wrote Excel functions to perform the calculation of population genetics parameters, as well as to test whether the population conforms to the HWE principle. Haplotype analysis was carried out using LDBlockShow (V1.40) software [14] to construct haplotype blocks to detect the region near the significant loci and whether they were in a highly interlocked block, and Haploview software [15] was used to conduct linkage disequilibrium analysis on SNPs of Inner Mongolia cashmere goats whose cashmere traits were significantly associated with HWE.One-way analysis of variance (ANOVA) was performed using SAS (V9.2) software [16], and the t-test was applied when only two genotypes were found in a particular locus, and phenotype-genotype association analysis was performed using SAS (V9.2) software, and the general linear model for the genotype-genotype association analysis was: y = u + G_i_ + E_ij_, with u being the overall mean, G_i_ representing the genotype effect, and E_ij_ representing the random error. Duncan's multiple comparisons were used to assess the significance of differences between genotypes (*P* < 0.05) and data were expressed as "mean ± standard error". Associations between the genotypes of 8 SNPs and cashmere traits were evaluated with a one-way analysis of variance (= three genotypes and sample size greater than or equal to 3) and independent sample t-test (= two genotypes) in SPSS Statistics (V25.0). When the analysis of variance performed on each group of genotypes indicated a significant difference (*P* < 0.05), statistical differences between the two genotypes were subsequently evaluated with the Bonferroni correction test.

## Results

### Estimation of genetic parameters of cashmere traits

The heritability of cashmere yield, cashmere diameter, and fleece length traits of Inner Mongolia cashmere goats were 0.229, 0.359, and 0.250, which belonged to the medium heritability traits (0.2 ~ 0.4). The cashmere thickness trait has a low heritability of 0.053 (Table 3). In addition, we calculated the repeatability of each trait. The repeatability of cashmere yield, cashmere diameter, and fleece length traits was higher: 0.372 ± 0.016, 0.555 ± 0.013, and 0.410 ± 0.018, respectively. The repeatability of the cashmere thickness character is low (0.207 ± 0.021). The genetic correlation between the cashmere yield trait and the other three traits was 0.212 ~ 0.835, all of which were positive. The genetic correlation between cashmere yield and cashmere thickness traits was as high as 0.835. The phenotypic correlation between the cashmere yield trait and the other 3 traits ranged from 0.168 to 0.290, all of which were positive. The phenotypic correlation between cashmere yield and cashmere thickness was the highest (0.290). The genetic correlation between cashmere diameter and cashmere thickness traits was positive (0.393), but the genetic correlation with fleece length traits was very low. Cashmere diameter was positively correlated with the phenotypic correlation of cashmere thickness and fleece length. The genetic and phenotypic correlations of cashmere thickness and fleece length traits were 0.475 and 0.252, respectively.

**Table 3:** The heritability of cashmere traits and the genetic and phenotypic correlations among traits in Inner Mongolia cashmere goats

Trait	Cashmere yield	Cashmere diameter	Cashmere thickness	Fleece length
Cashmere yield	**0.229** **(0.028)**	0.368 (0.032)	0.835 (0.047)	0.212 (0.041)
Cashmere diameter	0.203 (0.014)	**0.359** **(0.030)**	0.393 (0.051)	-0.001 (0.037)
Cashmere thickness	0.290 (0.013)	0.143 (0.015)	**0.053** **(0.020)**	0.475 (0.060)
Fleece length	0.168 (0.015)	0.030 (0.016)	0.252 (0.013)	**0.250** **(0.031)**

The upper triangle is genetic correlation (standard error), the lower triangle is phenotypic correlation (standard error), and the diagonal is heritability (standard error)

### Identification of genomic variations in Inner Mongolia cashmere goats

**Fig. 2:**
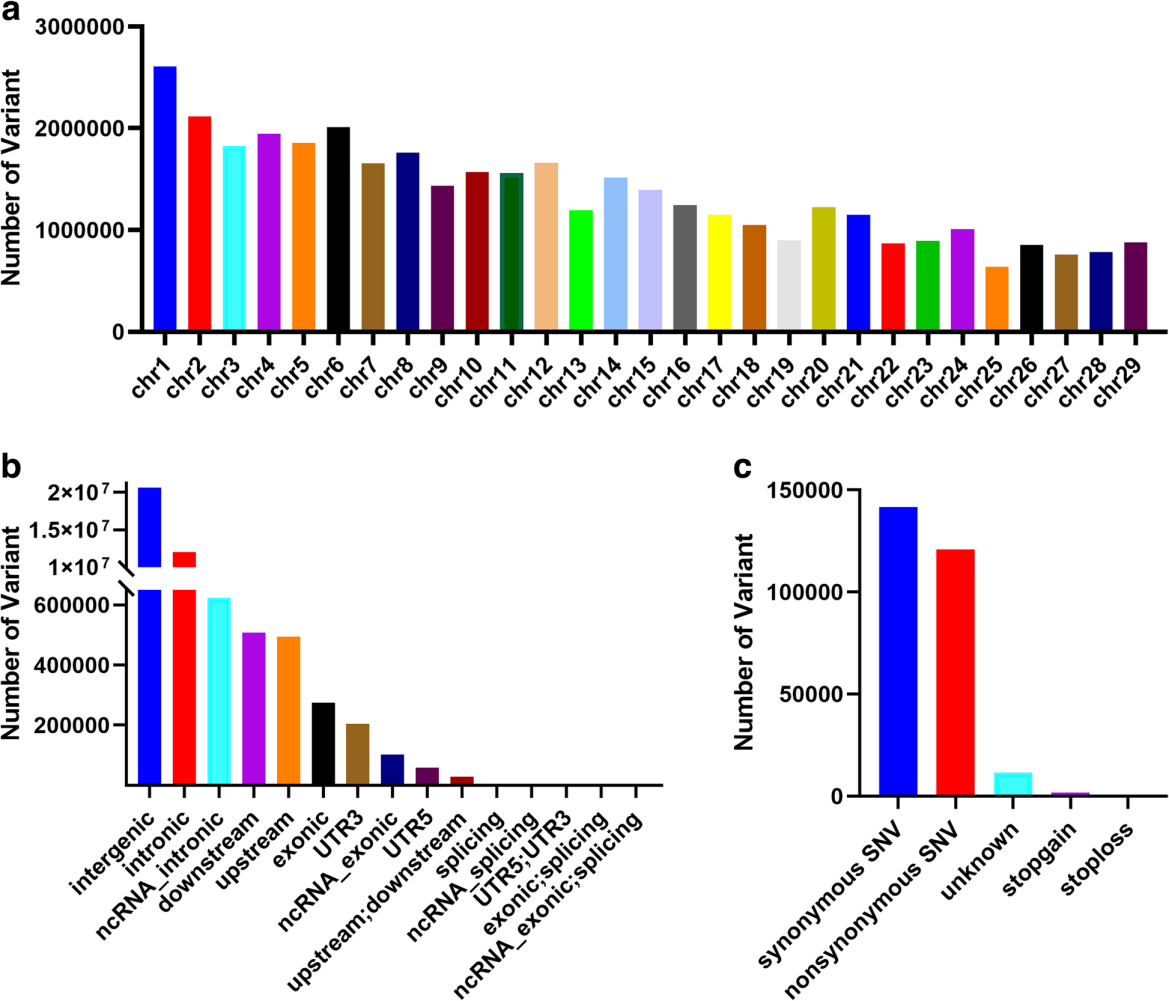
Variants characteristics of Inner Mongolia cashmere goat population. **a** Genome-wide distribution of detected variants on 29 chromosomes for Inner Mongolia cashmere goats. X-axis represents 29 autosomes; Y-axis represents the number of variants. **b** Genome-wide annotation of Inner Mongolia cashmere goats genetic variations. X-axis represents various functional regions; Y-axis represents the number of genetic variations within various functional regions. **c** Statistics of variants functional annotation results in CDS region. X-axis represents various functions; Y-axis represents the number of genetic variations within various functions

Whole genome resequencing was performed on 404 individuals of the Inner Mongolia cashmere goats (Erlangshan type), generating raw reads with a total size of 26,835.11 Gb. After variant detection and strict quality control, a total of 39,509,854 variants, including SNPs and Indels, were identified in the Inner Mongolia cashmere goat population (Fig. 2a). Then, all detected variants in Inner Mongolia cashmere goats were annotated using gene annotation files downloaded from the Ensembl database, and it was discovered that the highest number of variants were found in intergenic regions (59.09%) and intronic regions (34.34%) (Fig. 2b), of which only 0.79% were located in the coding regions, including 141,732 synonymous mutations and 120,751 non synonymous mutations (Fig. 2c). These potential functional variants provide valuable genetic resources for exploring the genetic structure and functional genes of Inner Mongolia cashmere goats.

### Data quality control, genetic relationship analysis and PCA analysis

**Fig. 3:**
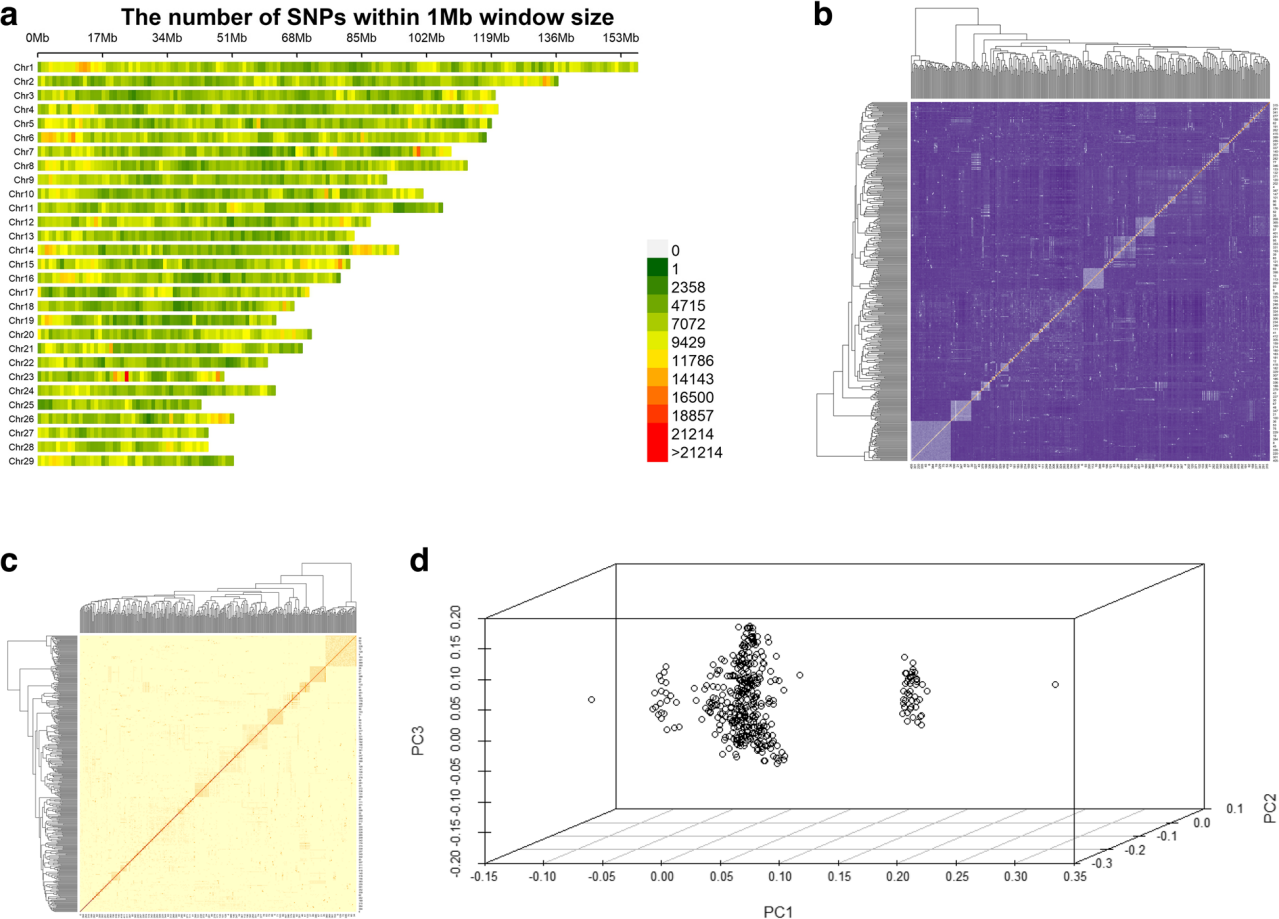
Visualization of SNPs on chromosomes, visualization of relatedness, and visualization of PCA. **a** Distribution of SNPs on chromosome 1 Mb window after quality control. The left Y-axis indicates the chromosome name and the upper X-axis indicates the window size. **b** Visualization of the IBS genetic distance matrix. Where each small square represents the value of genetic distance between the first to the last sample two by two, the larger the value the closer to purple, i.e., the larger the genetic distance between two individuals, the more distant the kinship, and vice versa. **c** G-matrix visualization. Each small square represents the kinship value between the first and last samples, the larger the value the closer to orange, i.e., the closer the two individuals are related and vice versa. **d** Visualization of PCA. The first three explain the variance percentage (PC1, PC2, and PC3) as the X, Y, and Z axes

A total of 34,248,064 SNPs were involved in quality control, 695,497 SNPs with a detection rate of less than 98% (–geno 0.02) were rejected, and the remaining loci were then filtered by HWE filtering, MAF filtering, and individual detection rate filtering (–maf 0.05, –hwe 1e-6, –mind 0.02), and a total of 17,135,082 SNPs loci were used for subsequent analysis, which were evenly distributed on 29 pairs of autosomes in goats (Fig. 3a).

Genetic distance analysis based on IBS and genomic genetic relationship analysis based on G-matrix could analyze the population without knowing the population lineage or ancestry samples. The results showed that the IBS distance values of the Inner Mongolia cashmere goat population ranged from 0.1298 to 0.2626, with an average genetic distance of 0.2400 ± 0.0111, indicating that the average genetic distance between individuals of Inner Mongolia cashmere goats was farther away and varied greatly. The visualization results of the IBS genetic distance matrix and G-matrix genetic relationship of the whole population are shown in Fig. 3b, c. Most of the individuals were moderately related to each other, while some of the cashmere goat individuals were more closely related to each other. The reason was the presence of both parents and their offspring in the sequenced individuals, which resulted in closer relatedness. We calculated the first three principal components by using Plink (V1.90) software, and the results showed that there was population stratification in the test sample (Fig. 3d). Therefore, we used "breeding value + residual" as the correction phenotype value to effectively correct the population stratification and ensure the accuracy of the experimental results.

### GWAS for cashmere traits and functional annotation of candidate genes

#### Cashmere yield

**Fig. 4:**
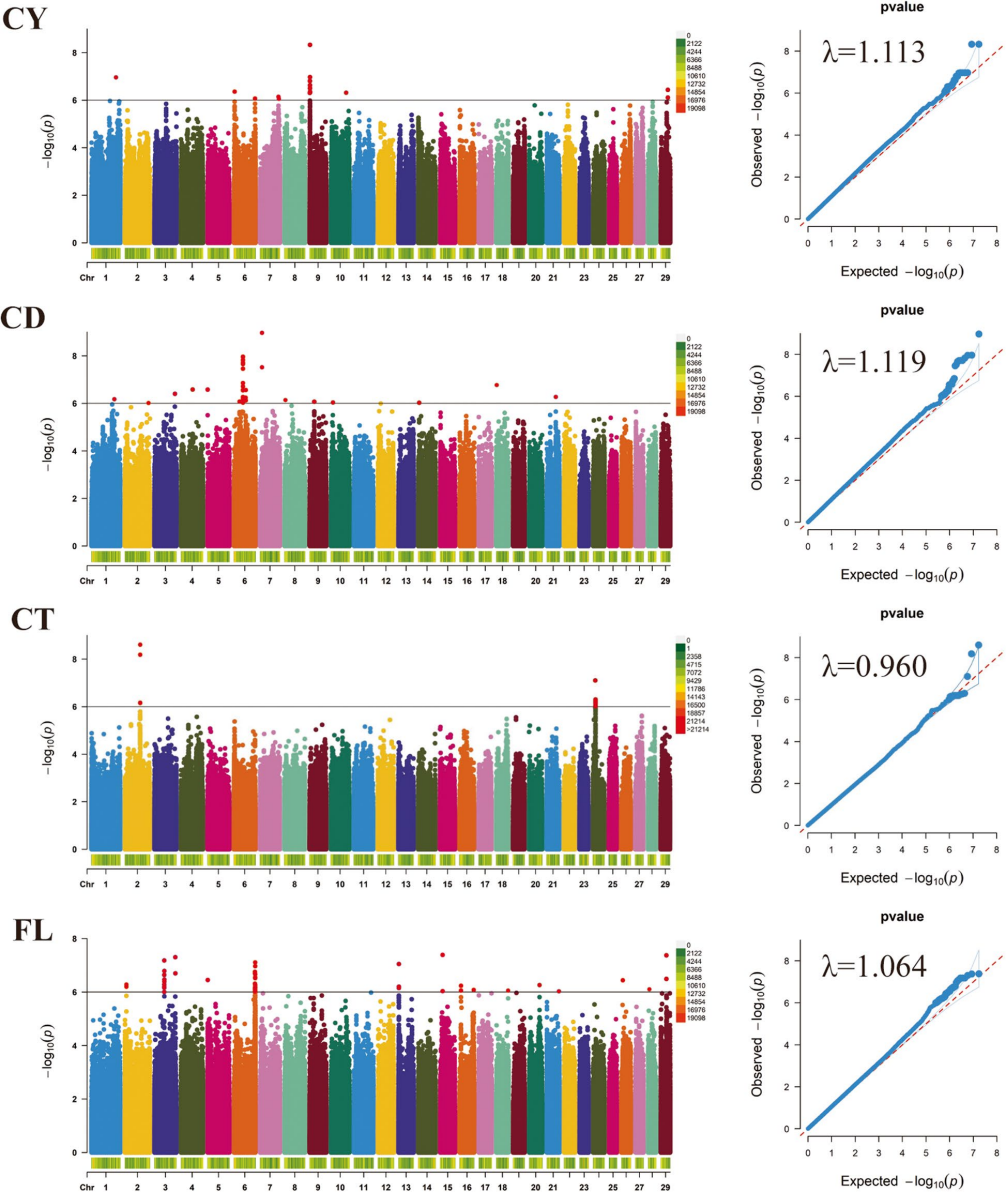
Manhattan Plots and QQ-plots showing GWAS results for four cashmere traits in Inner Mongolia cashmere goats. Genome-wide significant SNPs are shown in red.CY = cashmere yield, CD = cashmere diameter, CT = cashmere thickness, FL = fleece length

GWAS analysis of the cashmere yield trait based on resequencing data from 404 Inner Mongolia cashmere goats detected 28 significant SNPs (Fig. 4) according to the corrected threshold (*P* < 1 × 10^–6^), which were located on chromosomes 1, 6, 7, 9, 10, and 29, respectively (Table 4). Three SNPs on chromosome 7 annotated to the interior of the gene, chr7_g.100291226 T > C with chr7_g.100293016A > G located within the gene *LOC102170865*, and chr7_g.102631194A > G located within the gene *CPAMD8*; three SNPs on chromosome 9 (chr9_g.2058825G > A, chr9_g.2059470C > T, chr9_g.2059934 T > C) are annotated within *LOC102188675*; and chr10_g.82715068 T > C on chromosome 10 is annotated within *THSD4*.

**Table 4:** Description of significantly associated SNPs and potential candidate genes of cashmere traits in IMCGs

Traits	Number	SNP	BETA	*p* value	*r*^2^(%)	distance(bp)	Gene Name
Cashmere yield	SNP1	chr1_g.133340208 T > G	134.204	1.1002E-07	2.82	319,909	EPHB1
	SNP2	chr6_g.3398555C > T	97.5493	4.36438E-07	2.55	324,562	BBS7
	SNP3	chr6_g.114932667G > C	90.9386	8.53626E-07	2.42	-16,870	TRMT44
	SNP4	chr7_g.100291226 T > C	-92.098	7.17202E-07	2.46	within	LOC102170865
	SNP5	chr7_g.100293016A > G	-92.098	7.17202E-07	2.46	within	LOC102170865
	SNP6	chr7_g.100300867G > A	-92.098	7.17202E-07	2.46	6375	LOC108636448
	SNP7	chr7_g.100301174G > A	-92.098	7.17202E-07	2.46	6068	LOC108636448
	SNP8	chr7_g.102631194A > G	-74.902	8.75073E-07	2.42	within	CPAMD8
	SNP9	chr9_g.1037676A > C	-102.64	4.91578E-07	2.53	-	-
	SNP10	chr9_g.1038397C > T	-102.64	4.91578E-07	2.53	-	-
	SNP11	chr9_g.1322117C > A	-130.37	1.56229E-07	2.75	-	-
	SNP12	chr9_g.1328342G > A	-130.37	1.56229E-07	2.75	-	-
	SNP13	chr9_g.1622595C > G	-113.23	3.17596E-07	2.61	235,506	COL12A1
	SNP14	chr9_g.1624552 T > G	-116.18	1.06714E-07	2.83	233,549	COL12A1
	SNP15	chr9_g.1625830G > A	-115.22	2.36643E-07	2.67	232,271	COL12A1
	SNP16	chr9_g.1627029G > A	-116.18	1.06714E-07	2.83	231,072	COL12A1
	SNP17	chr9_g.1627149C > T	-122.83	4.71159E-09	3.43	230,952	COL12A1
	SNP18	chr9_g.1627193C > A	-122.83	4.71159E-09	3.43	230,952	COL12A1
	SNP19	chr9_g.1627936 T > C	-116.18	1.06714E-07	2.83	230,952	COL12A1
	SNP20	chr9_g.1627970 T > C	-115.22	2.36643E-07	2.67	230,952	COL12A1
	SNP21	chr9_g.1628474C > A	-116.18	1.06714E-07	2.83	230,952	COL12A1
	SNP22	chr9_g.2055327A > C	-111.65	4.46219E-07	2.55	2565	LOC102188675
	SNP23	chr9_g.2058825G > A	-112.12	4.74095E-07	2.54	within	LOC102188675
	SNP24	chr9_g.2059470C > T	-112.12	4.74095E-07	2.54	within	LOC102188675
	SNP25	chr9_g.2059934 T > C	-112.12	4.74095E-07	2.54	within	LOC102188675
	SNP26	chr10_g.82715068 T > C	-55.639	4.83934E-07	2.53	within	THSD4
	SNP27	chr29_g.38178615G > A	-126.22	7.77527E-07	2.44	-98,305	LOC102178345
	SNP28	chr29_g.38178652G > A	-131.17	3.66641E-07	2.59	-98,342	LOC102178345
Cashmere diameter	SNP29	chr1_g.124483769C > T	-0.2347	6.64118E-07	2.76	-16,300	C1H3orf58
	SNP30	chr2_g.128598723C > A	-0.4998	9.58969E-07	2.52	within	GULP1
	SNP31	chr3_g.111619565C > T	0.35956	3.94329E-07	2.40	19,728	F11R
	SNP32	chr4_g.63539664G > A	0.40299	2.60757E-07	2.49	-71,494	IMMP2L
	SNP33	chr5_g.82683A > G	-0.5486	2.61914E-07	2.79	-	-
	SNP34	chr6_g.30455840 T > C	0.51194	8.36107E-07	2.32	within	PDLIM5
	SNP35	chr6_g.30463541A > T	0.51194	8.36107E-07	2.32	within	PDLIM5
	SNP36	chr6_g.30464639A > G	0.51194	8.36107E-07	2.32	within	PDLIM5
	SNP37	chr6_g.30467066G > A	0.51194	8.36107E-07	2.32	within	PDLIM5
	SNP38	chr6_g.48834860C > T	-0.2423	8.69847E-07	2.69	-	-
	SNP39	chr6_g.48839975A > G	-0.2409	9.3506E-07	2.67	-	-
	SNP40	chr6_g.48845475C > A	-0.2819	2.21785E-08	3.52	-	-
	SNP41	chr6_g.48845609C > T	0.25353	7.24053E-07	2.24	-	-
	SNP42	chr6_g.48845801 T > A	-0.2837	1.93617E-08	3.55	-	-
	SNP43	chr6_g.48845806A > G	-0.2837	1.93617E-08	3.55	-	-
	SNP44	chr6_g.48850615G > A	0.27968	6.00824E-07	2.29	-	-
	SNP45	chr6_g.48850875G > C	0.29741	1.08073E-08	2.95	-	-
	SNP46	chr6_g.48851649 T > G	0.29612	1.12097E-08	2.94	-	-
	SNP47	chr6_g.48851736A > G	0.28969	2.82175E-07	2.42	-	-
	SNP48	chr6_g.48852274A > G	0.28999	2.00987E-08	2.84	-	-
	SNP49	chr6_g.48853045 T > C	0.28802	2.72224E-07	2.42	-	-
	SNP50	chr6_g.48854326G > A	0.27573	3.47602E-08	2.74	-	-
	SNP51	chr6_g.48854946G > T	0.29175	1.92579E-07	2.48	-	-
	SNP52	chr6_g.48854986A > G	0.2867	1.49704E-08	2.88	-	-
	SNP53	chr6_g.48855573A > G	0.29695	1.39633E-07	2.54	-	-
	SNP54	chr6_g.48856039A > G	0.28015	5.40789E-07	2.30	-	-
	SNP55	chr6_g.48857532 T > A	0.27912	5.99568E-07	2.29	-	-
	SNP56	chr6_g.63639667C > T	0.43598	5.77681E-07	2.36	within	KCTD8
	SNP57	chr6_g.64623978 T > C	0.39317	7.45874E-07	2.31	-	-
	SNP58	chr6_g.64624254G > A	0.41038	2.72887E-07	2.48	-	-
	SNP59	chr7_g.10569445C > T	-0.5874	1.07832E-09	3.97	within	ST8SIA4
	SNP60	chr7_g.10569447C > T	-0.5149	3.02897E-08	3.26	within	ST8SIA4
	SNP61	chr8_g.5264125A > G	0.51767	7.23629E-07	2.34	within	GALNTL6
	SNP62	chr9_g.25001979C > G	0.27484	8.52591E-07	2.23	-251,210	TRNAC-GCA-135
	SNP63	chr10_g.10834113C > G	0.2591	9.18427E-07	2.20	-573	TSHR
	SNP64	chr14_g.5228982C > G	0.27745	9.3957E-07	2.21	-58,823	LOC102174762
	SNP65	chr18_g.2201165C > A	0.48157	1.69188E-07	2.59	58,048	LOC102172289
	SNP66	chr21_g.49120096C > A	-0.5452	5.33483E-07	2.64	253,523	TRNAH-GUG-17
Cashmere thickness	SNP67	chr2_g.83535079 T > C	-0.439	6.53436E-09	3.65	within	GTDC1
	SNP68	chr2_g.83536939A > C	-0.4433	2.47683E-09	3.87	within	GTDC1
	SNP69	chr2_g.83793016C > T	-0.3951	6.8465E-07	2.63	within	GTDC1
	SNP70	chr2_g.83795309C > T	-0.3951	6.8465E-07	2.63	within	GTDC1
	SNP71	chr2_g.83801761 T > C	-0.4005	7.07375E-07	2.62	within	GTDC1
	SNP72	chr24_g.12811352C > T	-0.6421	7.92792E-08	3.02	-25,517	SYT4
	SNP73	chr24_g.14155537C > T	-0.3259	7.28123E-07	2.65	within	PIK3C3
	SNP74	chr24_g.14161470 T > A	-0.3298	5.00759E-07	2.74	within	PIK3C3
	SNP75	chr24_g.14162679G > C	-0.3255	6.83921E-07	2.67	within	PIK3C3
	SNP76	chr24_g.14163866C > T	-0.328	6.41421E-07	2.68	within	PIK3C3
	SNP77	chr24_g.14174933 T > C	-0.328	6.41421E-07	2.68	within	PIK3C3
	SNP78	chr24_g.14175220C > A	-0.328	6.41421E-07	2.68	within	PIK3C3
	SNP79	chr24_g.14175958A > C	-0.3212	9.72873E-07	2.59	within	PIK3C3
	SNP80	chr24_g.14180589C > T	-0.328	6.41421E-07	2.68	within	PIK3C3
	SNP81	chr24_g.14180758C > T	-0.328	6.41421E-07	2.68	within	PIK3C3
	SNP82	chr24_g.14189601C > T	-0.3277	5.6572E-07	2.71	within	PIK3C3
	SNP83	chr24_g.14194988A > G	-0.3246	8.87919E-07	2.61	within	PIK3C3
	SNP84	chr24_g.14195164A > C	-0.3304	5.49238E-07	2.71	within	PIK3C3
Fleece length	SNP85	chr2_g.8298760A > T	-1.2934	6.37E-07	2.53	within	RHCE
	SNP86	chr2_g.8302467 T > C	-1.2397	5.19E-07	2.57	within	RHCE
	SNP87	chr2_g.8302494A > G	-1.2369	6.03E-07	2.54	within	RHCE
	SNP88	chr2_g.8303830C > T	-1.2369	6.03E-07	2.54	within	RHCE
	SNP89	chr2_g.8303941 T > C	-1.2369	6.03E-07	2.54	within	RHCE
	SNP90	chr2_g.8303999 T > C	-1.2353	6.32E-07	2.53	within	RHCE
	SNP91	chr3_g.53642910 T > C	-0.7728	6.83606E-07	2.55	within	PIGK
	SNP92	chr3_g.53645354C > T	-0.781	6.3689E-07	2.56	within	PIGK
	SNP93	chr3_g.53645518G > T	-0.7771	9.90984E-07	2.47	within	PIGK
	SNP94	chr3_g.53647318C > T	-0.8478	6.58173E-08	3.02	within	PIGK
	SNP95	chr3_g.53647702 T > C	-0.8478	6.58173E-08	3.02	within	PIGK
	SNP96	chr3_g.53649661A > C	-0.7836	5.22181E-07	2.60	within	PIGK
	SNP97	chr3_g.53649874C > T	-0.8478	6.58173E-08	3.02	within	PIGK
	SNP98	chr3_g.53654279G > C	-0.8179	2.28585E-07	2.77	within	PIGK
	SNP99	chr3_g.53664849A > G	-0.8281	1.61465E-07	2.84	within	PIGK
	SNP100	chr3_g.53666146G > C	-0.7771	9.90984E-07	2.47	within	PIGK
	SNP101	chr3_g.53666620 T > A	-0.7771	9.90984E-07	2.47	within	PIGK
	SNP102	chr3_g.53668833C > T	-0.8056	3.40554E-07	2.69	within	PIGK
	SNP103	chr3_g.53670709A > C	-0.7973	4.02733E-07	2.65	within	PIGK
	SNP104	chr3_g.113687547A > G	1.89814	4.9777E-08	2.93	-88,971	CCDC190
	SNP105	chr3_g.114043472A > G	1.57668	2.00303E-07	2.66	-37,450	RGS5
	SNP106	chr5_g.685946 T > A	-0.8377	3.54689E-07	2.68	-10,990	TSPAN8
	SNP107	chr6_g.114105523C > T	0.94099	7.00175E-07	2.40	within	SORCS2
	SNP108	chr6_g.114106323C > T	0.91666	9.57523E-07	2.34	within	SORCS2
	SNP109	chr6_g.114106682G > T	0.92919	9.81067E-07	2.34	within	SORCS2
	SNP110	chr6_g.114108173C > T	0.93138	9.44323E-07	2.34	within	SORCS2
	SNP111	chr6_g.114108275G > A	0.82745	4.82585E-07	2.46	within	SORCS2
	SNP112	chr6_g.114111249A > G	0.80259	7.70025E-07	2.37	within	SORCS2
	SNP113	chr6_g.114111379G > A	0.85976	1.99487E-07	2.62	within	SORCS2
	SNP114	chr6_g.114111423A > G	0.8494	1.75194E-07	2.64	within	SORCS2
	SNP115	chr6_g.114111845G > A	0.8494	1.75194E-07	2.64	within	SORCS2
	SNP116	chr6_g.115574706C > T	-0.8242	8.67626E-07	2.49	within	RGS12
	SNP117	chr6_g.115578298C > G	-0.8702	7.79132E-08	2.98	within	RGS12
	SNP118	chr6_g.115578368G > A	-0.8202	5.52407E-07	2.59	within	RGS12
	SNP119	chr6_g.115578415A > C	-0.8488	2.91711E-07	2.71	within	RGS12
	SNP120	chr6_g.115578444G > A	-0.8277	6.62194E-07	2.55	within	RGS12
	SNP121	chr6_g.115581252G > A	-0.8371	5.36927E-07	2.59	within	RGS12
	SNP122	chr6_g.115581272C > A	-0.8216	6.90823E-07	2.54	within	RGS12
	SNP123	chr6_g.115583050G > T	-0.844	1.08123E-07	2.92	within	RGS12
	SNP124	chr6_g.115583302 T > C	-0.8163	9.36219E-07	2.48	within	RGS12
	SNP125	chr6_g.115583312G > A	-0.8163	9.36219E-07	2.48	within	RGS12
	SNP126	chr6_g.115584191 T > A	-0.863	2.8062E-07	2.72	within	RGS12
	SNP127	chr6_g.115586088G > C	-0.8678	2.16961E-07	2.77	within	RGS12
	SNP128	chr6_g.115586283G > A	-0.8536	2.83715E-07	2.72	within	RGS12
	SNP129	chr6_g.115599523 T > C	-0.7704	3.02844E-07	2.72	within	RGS12
	SNP130	chr6_g.115605297A > G	-0.7806	6.24597E-07	2.57	within	RGS12
	SNP131	chr6_g.115605508 T > C	-0.7795	8.99973E-07	2.49	within	RGS12
	SNP132	chr6_g.115606026 T > C	-0.7839	8.0574E-07	2.51	within	RGS12
	SNP133	chr6_g.115635769A > G	-0.8448	7.25243E-07	2.53	within	RGS12
	SNP134	chr13_g.2153013A > G	-0.8334	6.2975E-07	2.56	within	PLCB4
	SNP135	chr13_g.2153032 T > G	-0.8307	7.04463E-07	2.54	within	PLCB4
	SNP136	chr13_g.2153041 T > C	-0.8307	7.04463E-07	2.54	within	PLCB4
	SNP137	chr13_g.2153075 T > C	-0.883	8.94529E-08	2.95	within	PLCB4
	SNP138	chr15_g.13458287A > G	1.16007	4.08418E-08	2.93	-	-
	SNP139	chr15_g.13459723A > G	1.16381	9.22202E-07	2.36	-	-
	SNP140	chr16_g.7381769G > A	1.14888	8.7514E-07	2.37	-	-
	SNP141	chr16_g.7381773C > T	1.17253	5.81214E-07	2.45	-	-
	SNP142	chr16_g.76396893G > A	-1.5028	8.3369E-07	2.47	within	LOC102174324
	SNP143	chr18_g.64431725G > C	-1.3524	8.76882E-07	2.46	-10,161	LOC108638053
	SNP144	chr20_g.56722133C > T	1.44356	5.47209E-07	2.47	-94,187	ZNF622
	SNP145	chr21_g.65871544A > G	0.85105	9.38634E-07	2.34	65,591	PPP2R5C
	SNP146	chr26_g.7282942G > T	-1.0334	3.6172E-07	2.66	45,158	CTBP2
	SNP147	chr26_g.7284060 T > C	-1.0334	3.6172E-07	2.66	44,040	CTBP2
	SNP148	chr28_g.6796117G > C	-1.6127	7.87239E-07	2.48	-210,242	GHITM
	SNP149	chr28_g.6796120A > G	-1.6127	7.87239E-07	2.48	-210,245	GHITM
	SNP150	chr29_g.30412414G > A	1.3026	3.23263E-07	2.56	-	-
	SNP151	chr29_g.30414217 T > C	1.42037	4.23422E-08	2.94	-	-

r^2^ (%) represents the phenotypic variance explained by the SNP. Positive numbers in the distance column indicate the distance between the SNP and the gene upstream; negative numbers indicate the distance between the SNP and the gene downstream. In the table, only genes whose SNP are located within the gene or those closest to the SNP are shown as potential candidate genes. The full results of gene annotation are shown in Table S1

Among the 28 significantly associated SNPs, there were two SNPs that explained more than 3% of the phenotypic variation. The SNPs that explained the most phenotypic variation were chr9_g.1627149C > T and chr9_g.1627193C > A on chromosome 9, indicating a large contribution to the phenotype, and the phenotypic variation explained by these 2 SNPs was 3.43%, corresponding to the smallest, i.e., the most significant, *p*-value of 4.71 × 10^–9^, but the corresponding effect value was small, at -122.83. We believe that these 2 SNPs may be important genetic loci that play an important role in the growth and development of hair follicles in Inner Mongolia cashmere goats. The SNPs with the smallest explained phenotypic variation were chr7_g.102631194A > G, with the largest *p*-value of 8.75 × 10^–7^, corresponding to a larger effect value of -74.90.

#### Cashmere diameter

Genome-wide association study for the cashmere diameter trait was performed based on resequencing data from 404 Inner Mongolia cashmere goats, and 38 significant SNPs were detected according to the corrected threshold (*P* < 1 × 10^–6^) (Fig. 4), which were located on chromosomes 1, 2, 3, 4, 5, 6, 7, 8, 9, 10, 14, 18, and 21, respectively (Table 4). on chromosome 2 chr2_g.128598723C > A was annotated into the *GULP1* gene; 5 SNPs on chromosome 6 were annotated inside the gene, chr6_g.30455840 T > C, chr6_g.30463541A > T, chr6_g.30464639A > G, chr6_g.30467066G > A were annotated into the *PDLIM5* gene, and chr6_g.63639667C > T was annotated into the *KCTD8* gene; both SNPs on chromosome 7 were annotated into the *ST8SIA4* gene; and chr8_g.5264125A > G on chromosome 8 was annotated into the *GALNTL6* within the gene.

Among the 38 significantly associated SNPs, there were 5 SNPs that explained more than 3% of the phenotypic variation. The SNP with the largest explained phenotypic variation was chr7_g.10569445C > T on chromosome 7, which was able to explain 3.97% of the phenotypic variation, corresponding to the smallest, i.e., the most significant, *p*-value value of 1.08 × 10^–9^, corresponding to the smallest effect value of -0.59 among the 38 SNPs; and the SNP with the smallest explained phenotypic variation was chr10_g.10834113C > G, with the largest *p*-value of 9.18 × 10^–7^, but corresponding to a larger effect value of 0.26.

#### Cashmere thickness

Genome-wide association study of cashmere thickness traits was performed based on resequencing data from 404 Inner Mongolia cashmere goats, and 18 significant SNPs were detected according to the corrected threshold (*P* < 1 × 10^–6^) (Fig. 4), which were located on chromosomes 2 and 24, respectively (Table 4). all of the significant SNPs on chromosome 2 were annotated to the *GTDC1* gene; and all of the significant SNPs on chromosome 24, except chr24_g.12811352C > T, were annotated to the *PIK3C3* gene. The significantly associated SNPs on chromosome 2 were all annotated to the *GTDC1* gene; the significantly associated SNPs on chromosome 24 were all annotated to the *PIK3C3* gene, except for chr24_g.12811352C > T.

Among the 18 significantly associated SNPs, there were 3 SNPs that explained more than 3% of the phenotypic variance. The SNP with the largest explained phenotypic variation was chr2_g.83536939A > C on chromosome 2, which was able to explain 3.87% of the phenotypic variation, corresponding to the smallest, i.e., the most significant, *p*-value of 2.48 × 10^–9^, but corresponding to a small effect value of -0.44; the SNP with the smallest explained phenotypic variation was chr24_g.14175958A > C, with the largest *p*-value value of 9.73 × 10^–7^, and also corresponding to the largest effect value of -0.32.

#### Fleece length

Genome-wide association study of fleece length traits based on resequencing data from 404 Inner Mongolia cashmere goats detected 67 significant SNPs (Fig. 4) according to the corrected threshold (*P* < 1 × 10^–6^), which were located on chromosomes 2, 3, 5, 6, 13, 15, 16, 18, 20, 21, 26, 28, and 29, respectively (Table 4). 6 SNPs on chromosome 2 were annotated to the *RHCE* gene; 13 SNPs on chromosome 3 were annotated to the *PIGK* gene; and the first 6 SNPs on chromosome 6 were annotated to the *SORCS2* gene, the last 18 were annotated to the *RGS12* gene; the 4 SNPs on chromosome 13 were annotated to the *PLCB4* gene; the chr16_g.76396893G > A on chromosome 16 was annotated to within *LOC102174324*.

Among the 67 significantly associated SNPs, there were 3 SNPs that explained more than 3% of the phenotypic variation. The SNPs with the largest explained phenotypic variation were chr3_g.53647318C > T, chr3_g.53647702 T > C and chr3_g.53649874C > T on chromosome 3. The phenotypic variation that could be explained by these three SNPs was 3.02%, which corresponded to a smaller *p*-value of 6.58 × 10^–8^, corresponding to a small effect value of -0.85; the SNPs with the smallest explained phenotypic variation were chr6_g.114106682G > T on chromosome 6, with a smaller *p*-value value of 9.81 × 10^–7^, corresponding to a small effect value of 0.93.

### Candidate Gene GO functional annotation and KEGG enrichment analysis

**Fig. 5:**
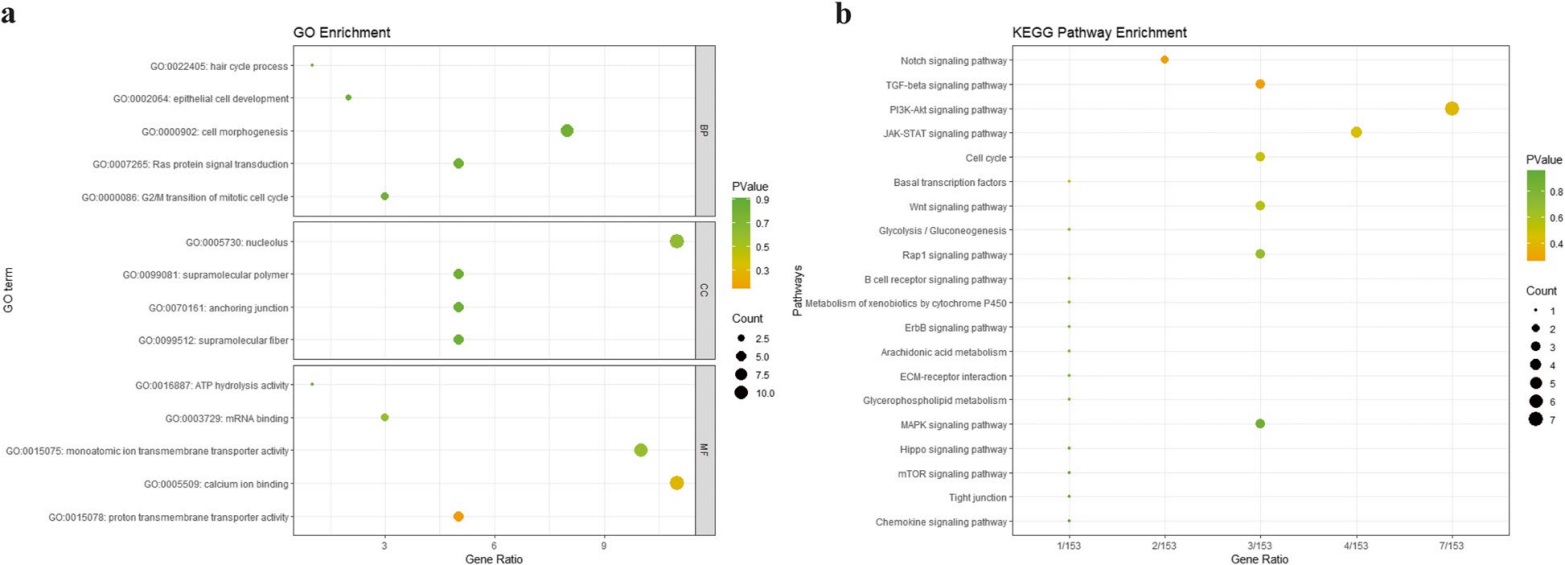
Bar chart of GO and KEGG enrichment analysis of the candidate genes. **a** represents the GO enrichment results, in the legend, BP represents biological process, CC represents cellular component, MF represents molecular function; **b** represents the KEGG enrichment results

We annotated 392 genes in 151 candidate regions and analyzed the candidate genes for GO functional annotation and KEGG enrichment using clusterProfiler package. The results obtained are shown in Table S2, S3, and these genes were enriched in 1674 GO and 209 KEGG pathways. The GO functional annotation results showed that these candidate genes were involved in the Biological Process of G2/M transition of mitotic cell cycle, Ras protein signal transduction, cell morphogenesis, epithelial cell development and hair cycle process, etc.; in terms of cellular component, they are involved in supramolecular fiber, anchoring junction, cell–cell junction, supramolecular polymer, nucleolus, etc.; in terms of molecular function, it is involved in proton transmembrane transporter activity, calcium ion binding, monoatomic ion transmembrane transporter activity, mRNA binding, ATP hydrolysis activity. For KEGG analysis, most of the important pathways are related to Environmental Information Processing, Cellular Processes and Metabolism, including Notch, P13K-Akt, TGF-beta, Cell cycle, Wnt, MAPK, Hippo, Ras signaling pathway, etc. (Fig. 5a, b).

### Population genetic parameters

Population genetic parameters of SNPs and potential candidate genes significantly associated with cashmere traits in Inner Mongoliacashmere goats are shown in Table 5. All 151 SNPs were quality controlled, and those with genotypic deletions were excluded, leaving 136 SNPs for subsequent analysis. Among them, 3 genotypes were detected for all 121 SNPs, of which 24 SNPs were significantly associated with cashmere yield, 27 SNPs were significantly associated with cashmere diameter, 17 SNPs were significantly associated with cashmere thickness, and 53 SNPs were significantly associated with fleece length; and 2 genotypes were detected for the other 15 SNPs, of which 4 SNPs were significantly associated with cashmere yield, 5 SNPs were significantly associated with cashmere diameter was significantly associated, and 6 SNPs were significantly associated with fleece length. The dominant genotypes of 92 SNPs were wild-type, and the dominant genotypes of 44 SNPs were heterozygous. The heterozygosity in this study is observed heterozygosity. It refers to the proportion of individuals in the population who are heterozygous at a certain site to the total number of individuals [17]. The lowest heterozygosity of 0.099 was found for chr1_g.133340208 T > G and chr21_g.49120096C > A, and the highest heterozygosity of 0.500 was found for chr1_g.124483769C > T. The effective allele numbers of the Inner Mongolia cashmere goat population ranged from 1.109 to 1.998. Polymorphic information content is related to the number and frequency of allele genes in a population, and is used to represent the degree of polymorphism at a certain site in a population to evaluate the genetic diversity of a population [18]. The polymorphic information content of 57 SNPs were all low polymorphism (0 < PIC < 0.25), and the polymorphic information content of 79 SNPs were all moderate polymorphism (0.25 < PIC < 0.50). It indicates that the genetic diversity of Inner Mongolia cashmere goat population is relatively high. The chi-square test found that 26 SNPs significantly deviated from the HWE (*P* < 0.05) and were not available for subsequent analysis; 110 SNPs conformed to the HWE (*P* > 0.05) and were available for subsequent analysis.

**Table 5:** Population genetic parameters of significantly associated SNPs and potential candidate genes of cashmere traits in IMCGs

Traits	Number	SNP^a^	HWE^b^		*Ho*^c^	*He*^d^	*PIC*^e^	*Ne*^f^
			***χ***^***2***^	***P***				
Cashmere yield	SNP1	chr1_g.133340208 T > G	0.841	0.359	0.901	0.099	0.094	1.109
	SNP2	chr6_g.3398555C > T	0.401	0.526	0.818	0.182	0.166	1.223
	SNP3	chr6_g.114932667G > C	0.879	0.348	0.816	0.184	0.167	1.226
	SNP4	chr7_g.100291226 T > C	1.217	0.27	0.798	0.202	0.181	1.253
	SNP5	chr7_g.100293016A > G	1.217	0.27	0.798	0.202	0.181	1.253
	SNP6	chr7_g.100300867G > A	1.217	0.27	0.798	0.202	0.181	1.253
	SNP7	chr7_g.100301174G > A	1.217	0.27	0.798	0.202	0.181	1.253
	SNP8	chr7_g.102631194A > G	0.387	0.534	0.707	0.293	0.25	1.414
	SNP9	chr9_g.1037676A > C	0	0.984	0.842	0.158	0.146	1.188
	SNP10	chr9_g.1038397C > T	0	0.984	0.842	0.158	0.146	1.188
	SNP11	chr9_g.1322117C > A	1.684	0.194	0.886	0.114	0.107	1.129
	SNP12	chr9_g.1328342G > A	1.684	0.194	0.886	0.114	0.107	1.129
	SNP13	chr9_g.1622595C > G	0.668	0.414	0.871	0.129	0.121	1.148
	SNP14	chr9_g.1624552 T > G	0.47	0.493	0.867	0.133	0.124	1.154
	SNP15	chr9_g.1625830G > A	0.783	0.376	0.873	0.127	0.119	1.145
	SNP16	chr9_g.1627029G > A	0.47	0.493	0.867	0.133	0.124	1.154
	SNP17	chr9_g.1627149C > T	1.296	0.255	0.858	0.142	0.132	1.165
	SNP18	chr9_g.1627193C > A	1.296	0.255	0.858	0.142	0.132	1.165
	SNP19	chr9_g.1627936 T > C	0.47	0.493	0.867	0.133	0.124	1.154
	SNP20	chr9_g.1627970 T > C	0.783	0.376	0.873	0.127	0.119	1.145
	SNP21	chr9_g.1628474C > A	0.47	0.493	0.867	0.133	0.124	1.154
	SNP22	chr9_g.2055327A > C	0.013	0.91	0.865	0.135	0.126	1.157
	SNP23	chr9_g.2058825G > A	0.004	0.951	0.867	0.133	0.124	1.154
	SNP24	chr9_g.2059470C > T	0.004	0.951	0.867	0.133	0.124	1.154
	SNP25	chr9_g.2059934 T > C	0.004	0.951	0.867	0.133	0.124	1.154
	SNP26	chr10_g.82715068 T > C	3.212	0.073	0.505	0.495	0.372	1.979
	SNP27	chr29_g.38178615G > A	1.472	0.225	0.893	0.107	0.102	1.12
	SNP28	chr29_g.38178652G > A	1.405	0.236	0.895	0.105	0.1	1.118
Cashmere diameter	SNP29	chr1_g.124483769C > T	0.047	0.828	0.5	0.5	0.375	1.998
	SNP30	chr2_g.128598723C > A	0.082	0.774	0.893	0.107	0.102	1.12
	SNP31	chr3_g.111619565C > T	15.127	0	0.728	0.272	0.235	1.373
	SNP32	chr4_g.63539664G > A	7.172	0.007	0.792	0.208	0.186	1.262
	SNP34	chr6_g.30455840 T > C	0.037	0.848	0.897	0.103	0.098	1.115
	SNP35	chr6_g.30463541A > T	0.037	0.848	0.897	0.103	0.098	1.115
	SNP36	chr6_g.30464639A > G	0.037	0.848	0.897	0.103	0.098	1.115
	SNP37	chr6_g.30467066G > A	0.037	0.848	0.897	0.103	0.098	1.115
	SNP38	chr6_g.48834860C > T	3.332	0.068	0.501	0.499	0.375	1.997
	SNP39	chr6_g.48839975A > G	2.964	0.085	0.501	0.499	0.375	1.997
	SNP40	chr6_g.48845475C > A	6.246	0.012	0.505	0.495	0.373	1.982
	SNP41	chr6_g.48845609C > T	2.049	0.152	0.547	0.453	0.35	1.828
	SNP42	chr6_g.48845801 T > A	6.702	0.01	0.504	0.496	0.373	1.982
	SNP43	chr6_g.48845806A > G	6.702	0.01	0.504	0.496	0.373	1.982
	SNP44	chr6_g.48850615G > A	6.539	0.011	0.598	0.402	0.321	1.672
	SNP45	chr6_g.48850875G > C	13.797	0	0.501	0.499	0.375	1.997
	SNP46	chr6_g.48851649 T > G	13.036	0	0.501	0.499	0.375	1.998
	SNP47	chr6_g.48851736A > G	7.527	0.006	0.599	0.401	0.32	1.669
	SNP48	chr6_g.48852274A > G	12.3	0	0.501	0.499	0.375	1.998
	SNP49	chr6_g.48853045 T > C	6.226	0.013	0.599	0.401	0.32	1.669
	SNP50	chr6_g.48854326G > A	4.879	0.027	0.505	0.495	0.373	1.981
	SNP51	chr6_g.48854946G > T	6.539	0.011	0.598	0.402	0.321	1.672
	SNP52	chr6_g.48854986A > G	6.738	0.009	0.507	0.493	0.372	1.973
	SNP53	chr6_g.48855573A > G	7.195	0.007	0.6	0.4	0.32	1.666
	SNP54	chr6_g.48856039A > G	4.509	0.034	0.606	0.394	0.316	1.65
	SNP55	chr6_g.48857532 T > A	4.776	0.029	0.605	0.395	0.317	1.653
	SNP56	chr6_g.63639667C > T	0.19	0.663	0.858	0.142	0.132	1.165
	SNP61	chr8_g.5264125A > G	1.472	0.225	0.893	0.107	0.102	1.12
	SNP62	chr9_g.25001979C > G	3.066	0.08	0.613	0.387	0.312	1.632
	SNP64	chr14_g.5228982C > G	2.956	0.086	0.624	0.376	0.305	1.603
	SNP65	chr18_g.2201165C > A	2.791	0.095	0.858	0.142	0.132	1.165
	SNP66	chr21_g.49120096C > A	1.215	0.27	0.901	0.099	0.094	1.109
Cashmere thickness	SNP67	chr2_g.83535079 T > C	0.013	0.909	0.656	0.344	0.285	1.523
	SNP68	chr2_g.83536939A > C	0.244	0.621	0.652	0.348	0.287	1.533
	SNP69	chr2_g.83793016C > T	0	0.99	0.681	0.319	0.268	1.469
	SNP70	chr2_g.83795309C > T	0	0.99	0.681	0.319	0.268	1.469
	SNP72	chr24_g.12811352C > T	0.071	0.79	0.858	0.142	0.132	1.165
	SNP73	chr24_g.14155537C > T	0.625	0.429	0.514	0.486	0.368	1.945
	SNP74	chr24_g.14161470 T > A	0.499	0.48	0.515	0.485	0.368	1.943
	SNP75	chr24_g.14162679G > C	0.45	0.502	0.514	0.486	0.368	1.946
	SNP76	chr24_g.14163866C > T	0.766	0.381	0.514	0.486	0.368	1.946
	SNP77	chr24_g.14174933 T > C	0.766	0.381	0.514	0.486	0.368	1.946
	SNP78	chr24_g.14175220C > A	0.766	0.381	0.514	0.486	0.368	1.946
	SNP79	chr24_g.14175958A > C	0.387	0.534	0.515	0.485	0.367	1.942
	SNP80	chr24_g.14180589C > T	0.766	0.381	0.514	0.486	0.368	1.946
	SNP81	chr24_g.14180758C > T	0.766	0.381	0.514	0.486	0.368	1.946
	SNP82	chr24_g.14189601C > T	0.45	0.502	0.514	0.486	0.368	1.946
	SNP83	chr24_g.14194988A > G	0.922	0.337	0.513	0.487	0.368	1.948
	SNP84	chr24_g.14195164A > C	0.922	0.337	0.513	0.487	0.368	1.948
Fleece length	SNP85	chr2_g.8298760A > T	1.321	0.25	0.849	0.151	0.14	1.178
	SNP87	chr2_g.8302494A > G	1.007	0.316	0.818	0.182	0.166	1.223
	SNP88	chr2_g.8303830C > T	1.007	0.316	0.818	0.182	0.166	1.223
	SNP89	chr2_g.8303941 T > C	1.007	0.316	0.818	0.182	0.166	1.223
	SNP91	chr3_g.53642910 T > C	3.686	0.055	0.557	0.443	0.345	1.796
	SNP92	chr3_g.53645354C > T	2.14	0.144	0.554	0.446	0.346	1.804
	SNP93	chr3_g.53645518G > T	4.349	0.037	0.577	0.423	0.333	1.732
	SNP94	chr3_g.53647318C > T	3.761	0.052	0.564	0.436	0.341	1.772
	SNP95	chr3_g.53647702 T > C	3.761	0.052	0.564	0.436	0.341	1.772
	SNP96	chr3_g.53649661A > C	3.426	0.064	0.558	0.442	0.344	1.791
	SNP97	chr3_g.53649874C > T	3.761	0.052	0.564	0.436	0.341	1.772
	SNP98	chr3_g.53654279G > C	5.036	0.025	0.576	0.424	0.334	1.735
	SNP99	chr3_g.53664849A > G	5.036	0.025	0.576	0.424	0.334	1.735
	SNP100	chr3_g.53666146G > C	4.349	0.037	0.577	0.423	0.333	1.732
	SNP101	chr3_g.53666620 T > A	4.349	0.037	0.577	0.423	0.333	1.732
	SNP102	chr3_g.53668833C > T	5.386	0.02	0.577	0.423	0.333	1.732
	SNP103	chr3_g.53670709A > C	5.769	0.016	0.576	0.424	0.334	1.738
	SNP104	chr3_g.113687547A > G	1.34	0.247	0.897	0.103	0.098	1.115
	SNP105	chr3_g.114043472A > G	0.652	0.419	0.867	0.133	0.124	1.154
	SNP106	chr5_g.685946 T > A	2.723	0.099	0.526	0.474	0.361	1.899
	SNP107	chr6_g.114105523C > T	1.799	0.18	0.65	0.35	0.289	1.539
	SNP108	chr6_g.114106323C > T	0.347	0.556	0.654	0.346	0.286	1.53
	SNP109	chr6_g.114106682G > T	1.496	0.221	0.652	0.348	0.287	1.533
	SNP110	chr6_g.114108173C > T	1.219	0.27	0.655	0.345	0.285	1.527
	SNP111	chr6_g.114108275G > A	1.189	0.276	0.54	0.46	0.354	1.852
	SNP112	chr6_g.114111249A > G	0.89	0.346	0.532	0.468	0.359	1.88
	SNP113	chr6_g.114111379G > A	1.707	0.191	0.54	0.46	0.354	1.852
	SNP114	chr6_g.114111423A > G	1.044	0.307	0.531	0.469	0.359	1.882
	SNP115	chr6_g.114111845G > A	1.044	0.307	0.531	0.469	0.359	1.882
	SNP116	chr6_g.115574706C > T	0.152	0.696	0.573	0.427	0.336	1.746
	SNP119	chr6_g.115578415A > C	0.232	0.63	0.56	0.44	0.343	1.786
	SNP120	chr6_g.115578444G > A	0.501	0.479	0.56	0.44	0.343	1.786
	SNP121	chr6_g.115581252G > A	0.001	0.973	0.577	0.423	0.333	1.732
	SNP122	chr6_g.115581272C > A	0.232	0.63	0.56	0.44	0.343	1.786
	SNP123	chr6_g.115583050G > T	0.081	0.776	0.541	0.459	0.354	1.85
	SNP124	chr6_g.115583302 T > C	0.845	0.358	0.555	0.445	0.346	1.802
	SNP125	chr6_g.115583312G > A	0.845	0.358	0.555	0.445	0.346	1.802
	SNP126	chr6_g.115584191 T > A	0.455	0.5	0.569	0.431	0.338	1.758
	SNP130	chr6_g.115605297A > G	0.126	0.723	0.529	0.471	0.36	1.891
	SNP131	chr6_g.115605508 T > C	0	0.989	0.532	0.468	0.358	1.878
	SNP132	chr6_g.115606026 T > C	0.009	0.926	0.532	0.468	0.359	1.88
	SNP133	chr6_g.115635769A > G	0.001	0.978	0.596	0.404	0.322	1.678
	SNP134	chr13_g.2153013A > G	1.651	0.199	0.551	0.449	0.348	1.815
	SNP135	chr13_g.2153032 T > G	1.844	0.174	0.55	0.45	0.349	1.818
	SNP136	chr13_g.2153041 T > C	1.844	0.174	0.55	0.45	0.349	1.818
	SNP137	chr13_g.2153075 T > C	1.515	0.218	0.541	0.459	0.354	1.85
	SNP138	chr15_g.13458287A > G	0.016	0.9	0.738	0.262	0.227	1.354
	SNP139	chr15_g.13459723A > G	0.872	0.35	0.8	0.2	0.18	1.25
	SNP140	chr16_g.7381769G > A	14.051	0	0.735	0.265	0.23	1.36
	SNP141	chr16_g.7381773C > T	13.532	0	0.738	0.262	0.227	1.354
	SNP142	chr16_g.76396893G > A	2.599	0.107	0.863	0.137	0.128	1.159
	SNP144	chr20_g.56722133C > T	1.265	0.261	0.85	0.15	0.139	1.177
	SNP145	chr21_g.65871544A > G	0.259	0.611	0.599	0.401	0.32	1.669
	SNP146	chr26_g.7282942G > T	0.552	0.457	0.725	0.275	0.237	1.379
	SNP147	chr26_g.7284060 T > C	0.552	0.457	0.725	0.275	0.237	1.379
	SNP148	chr28_g.6796117G > C	1.834	0.176	0.882	0.118	0.111	1.134
	SNP149	chr28_g.6796120A > G	1.834	0.176	0.882	0.118	0.111	1.134
	SNP150	chr29_g.30412414G > A	0.604	0.437	0.812	0.188	0.171	1.232
	SNP151	chr29_g.30414217 T > C	0.342	0.559	0.82	0.18	0.164	1.22

^a^*NSNP* single nucleotide polymorphism

^b^*HWE* Hardy–Weinberg Equilibrium test

^c^*Ho* Homozygosity

^d^*He* Heterozygosity

^e^*PIC* Polymorphism information content

^f^*Ne* Effective allele numbers

### Association analysis of SNPs polymorphisms with phenotypic values of cashmere traits

**Fig. 6:**
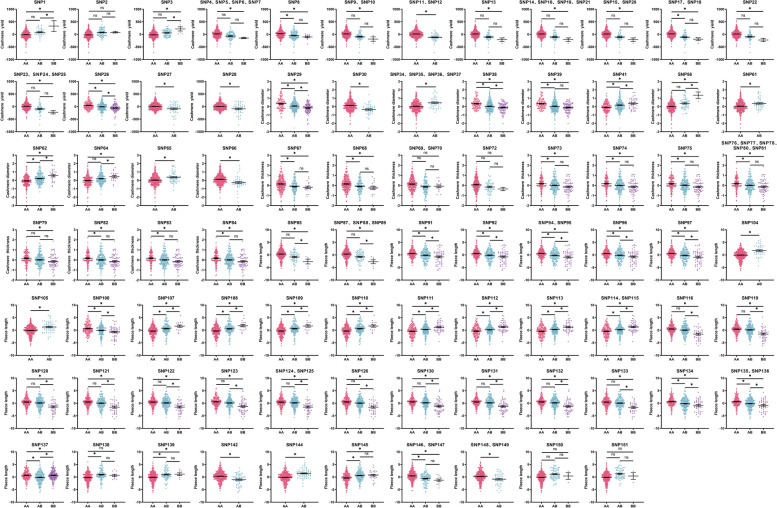
Assocaition analysis of significantly associated SNPs with phenotypes of cashmere traits in IMCGs. 110 SNPs conforming to the HWE were analyzed. "*" indicates a significant difference, and "ns" indicates insignificant difference

The 110 SNPs mentioned above that were conform for HWE were analyzed for association with cashmere traits, and the results of the association analysis of SNPs significantly associated with cashmere traits and potential candidate genes with the phenotypic values of cashmere traits in Inner Mongolia cashmere goats are shown in Fig. 6 and Table S4. 17 SNPs (SNP4, SNP5, SNP6, SNP7, SNP9, SNP10, SNP13, SNP14, SNP15, SNP16, SNP19, SNP20, SNP21, SNP22, SNP23, SNP24, SNP25) wild-type were significantly higher than those of the homozygous mutant (*P* < 0.05); SNP8, SNP17 and SNP18 wild type were significantly higher than those of the heterozygous mutant and the homozygous mutant (*P* < 0.05); SNP11, SNP12, SNP27 and SNP28 wild types were significantly higher than heterozygous mutants (*P* < 0.05); the cashmere yield of SNP1 and SNP3 homozygous mutants was significantly higher than that of the wild type and the heterozygous mutants (*P* < 0.05); moreover, the homozygous mutant types of these 2 SNPs were associated with the highest cashmere yield; and the cashmere yield of SNP26 wild type was significantly higher than the heterozygous mutant (*P* < 0.05), and the heterozygous mutant had significantly higher cashmere yield than the homozygous mutant (*P* < 0.05).

SNP29 and SNP38 homozygous mutants had significantly better cashmere diameter than heterozygous mutants (*P* < 0.05) and heterozygous mutants were significantly better than wild type (*P* < 0.05); SNP30 and SNP66 heterozygous mutants had significantly better cashmere diameter than wild type individuals (*P* < 0.05) and heterozygous mutants of SNP30 were associated with the lowest cashmere diameter; SNP34, SNP35, SNP36, SNP37, SNP61, and SNP65 wild type were significantly better than heterozygous mutants (*P* < 0.05); SNP39 homozygous and heterozygous mutants were significantly better cashmere diameter than wild type individuals (*P* < 0.05), while there was no significant difference between homozygous and heterozygous mutant individuals (*P* > 0.05); The cashmere diameter of SNP41 and SNP62 wild type was significantly better than heterozygous mutant (*P* < 0.05), and heterozygous mutant was significantly better than homozygous mutant (*P* < 0.05); the cashmere diameter of wild type and heterozygous mutant at SNP56 and SNP64 was significantly better than that of homozygous mutant (*P* < 0.05), and the cashmere diameter of wild type was better than heterozygous mutant but the difference was not significant (*P* > 0.05).

The cashmere thickness of the wild type of 13 SNPs (SNP67, SNP68, SNP73, SNP74, SNP75, SNP76, SNP77, SNP78, SNP80, SNP81, SNP82, SNP83, SNP84) was significantly higher than that of the heterozygous mutant and the homozygous mutant (*P* < 0.05) and there was no significant difference between the heterozygous mutant and the homozygous mutant individuals were not significantly different from each other (*P* > 0.05), the wild types of SNP76, SNP77, SNP78, SNP80, and SNP81 were associated with the highest cashmere thickness; the cashmere thickness of SNP79 wild type individuals was significantly higher than that of the homozygous mutant type (*P* < 0.05), and the differences among the genotypes of SNP69, SNP70, and SNP72 were insignificant (*P* > 0.05).

Seventeen SNPs (SNP85, SNP87, SNP88, SNP89, SNP116, SNP119, SNP120, SNP121, SNP122, SNP123, SNP124, SNP125, SNP126, SNP130, SNP131, SNP132, and SNP133) wild type and heterozygous mutant had significantly higher cashmere length than the homozygous mutant (*P* < 0.05), and the wild type was higher than the heterozygous mutant but there was no significant difference (*P* > 0.05); 11 SNPs (SNP91, SNP92, SNP94, SNP95, SNP96, SNP97, SNP106, SNP134, SNP135, SNP136, SNP137) wild type had significantly higher fleece length than heterozygous mutants (*P* < 0.05) and heterozygous mutants had significantly higher fleece length than homozygous mutants (*P* < 0.05), and the wild type of SNP137 was associated with the highest fleece length; 9 SNPs (SNP107, SNP108, SNP109, SNP110, SNP111, SNP112 SNP113, SNP114, SNP115) had significantly higher fleece lengths in the homozygous mutant than in the heterozygous mutant (*P* < 0.05), while the heterozygous mutant had significantly higher fleece lengths than the wild type (*P* < 0.05); SNP104, SNP105, SNP138, and SNP144 heterozygous mutants had significantly higher fleece lengths than the wild type (*P* < 0.05); SNP139 and SNP145 homozygous and heterozygous mutants had significantly higher fleece length than wild type (*P* < 0.05), and the fleece length of homozygous mutants was higher than that of heterozygous mutants but the difference between the two was not significant (*P* > 0.05); the fleece length of wild type individuals of SNP142, SNP148 and SNP149 was significantly higher than that of heterozygous mutants (*P* < 0.05); SNP146 and SNP147 wild type fleece length was significantly higher than heterozygous mutant and homozygous mutant (*P* < 0.05), and heterozygous mutant was higher than homozygous mutant fleece length but the difference between them was not significant (*P* > 0.05).

### Haplotype block analysis

Since most of the significant SNPs on chromosomes 2, 3, 6, 7, 9, 13, 16, and 29 are in close proximity to each other and may have strong linkage disequilibrium, the 151 SNPs that were significantly associated with the cashmere traits were analyzed using the LDBlockShow (v1.40) software in accordance with the D' > 0.33, *r*^2^ > 0.1 [19] to construct haplotype blocks on a genome wide scale. The SNPs significantly associated with cashmere yield formed 4 blocks with block, with block sizes ranging from 0.04 kb to 5.88 kb (Table S5, Fig. S1); The SNPs significantly associated with cashmere diameter formed 4 blocks with block sizes ranging from 3.20 kb-12.05 kb (Table S5, Fig. S2); The SNPs significantly associated with cashmere thickness formed a block with a block size of 1.86 kb (Table S5, Fig. S3); The SNPs significantly associated with fleece length formed 12 blocks, with block sizes ranging from 0.01 kb to 26.54 kb (Table S5, Fig. S4).

### Linkage disequilibrium analysis

**Fig. 7:**
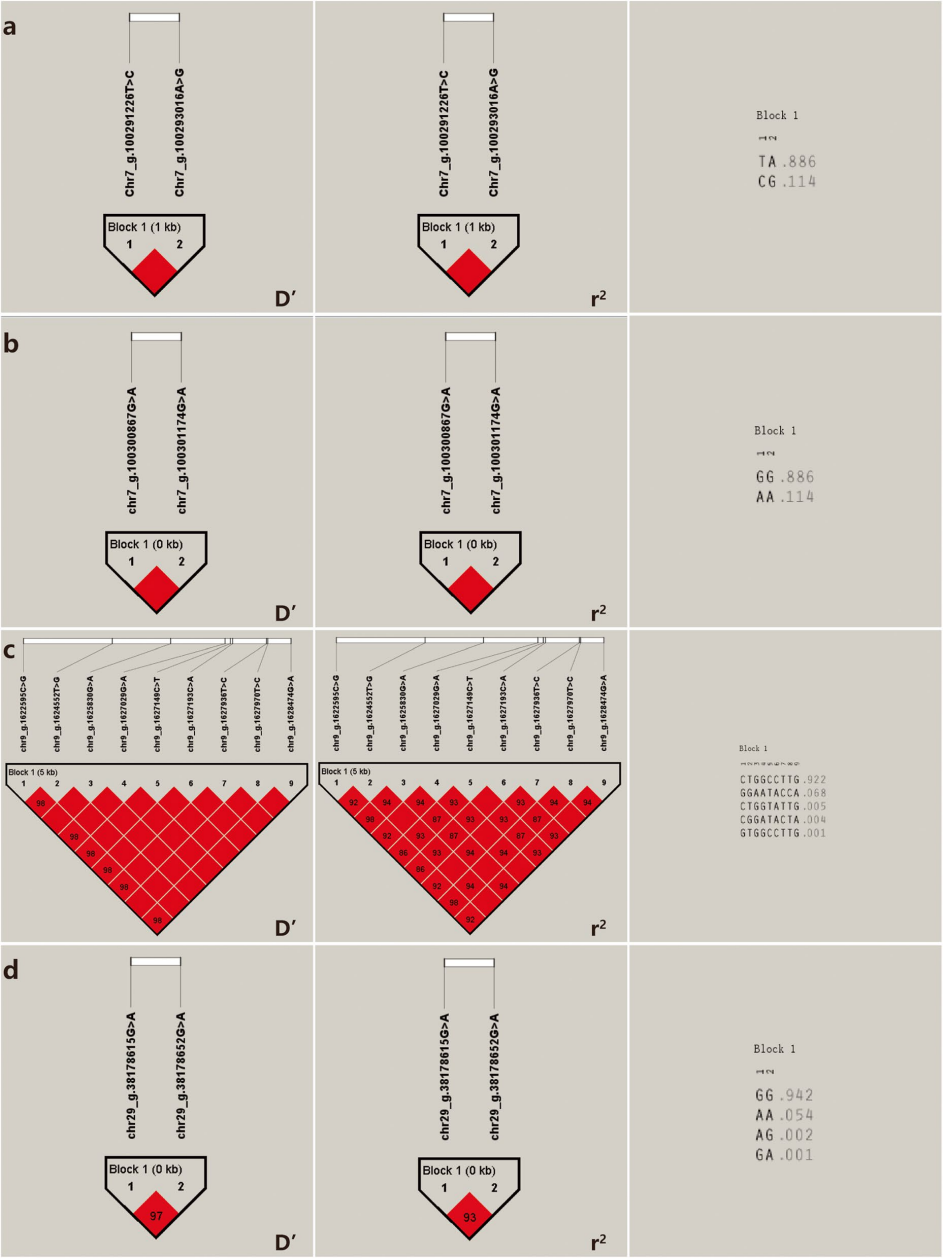
Results of linkage disequilibrium analysis and haplotype block of significantly associated SNPs of cashmere yield in IMCGs. **a** D' value, *r*^2^ value and haplotype frequency of block formed by 2 SNPs in the *LOC102170865* gene. **b** D' value, *r*^2^ value and haplotype frequency of block formed by 2 SNPs in the *LOC108636448* gene. **c** D' value, *r*^2^ value and haplotype frequency of block formed by 9 SNPs in the *COL12A1* gene. **d** D' value, *r*^2^ value and haplotype frequency of block formed by 2 SNPs in the *LOC102178345* gene. The linkage between loci was judged by D' and *r*^2^ values, where D' = 1 or *r*^2^ = 1 is called full linkage, D' = 0 or *r*^2^ = 0 is no linkage or linkage equilibrium, D' > 0.80 and *r*^2^ > 0.33 suggests strong linkage. The following is the same as in the previous sentence

Based on the above screening results, Haploview software was used to analyze the linkage disequilibrium of SNPs that were significantly associated with cashmere traits in Inner Mongolia cashmere goats and conformed HWE. The 15 SNPs significantly associated with cashmere yield traits in Inner Mongolia cashmere goats constituted 4 Blocks (Fig. 7), and there was a complete linkage (D' = 1) between chr7_g.100291226 T > C and chr7_g.100293016A > G of *LOC102170865* gene; complete linkage (D' = 1) between chr7_g.100300867G > A and chr7_g.100301174G > A of *LOC108636448* gene. There was a strong linkage disequilibrium (D' > 0.8 and r^2^ > 0.33) between chr9_g.1622595C > G and chr9_g.1624552 T > G, chr9_g.1627029G > A, chr9_g.1627149C > T, chr9_g.1627193C > A, chr9_g.1627936 T > C, chr9_ g.1628474C > A of *COL12A1* gene, and complete linkage (D' = 1) between other SNPs. chr29_ g.38178615G > A and chr29_g.38178652G > A have strong linkage disequilibrium (D' > 0.8 and *r*^2^ > 0.33) between them of *LOC102178345* gene.

**Fig. 8:**
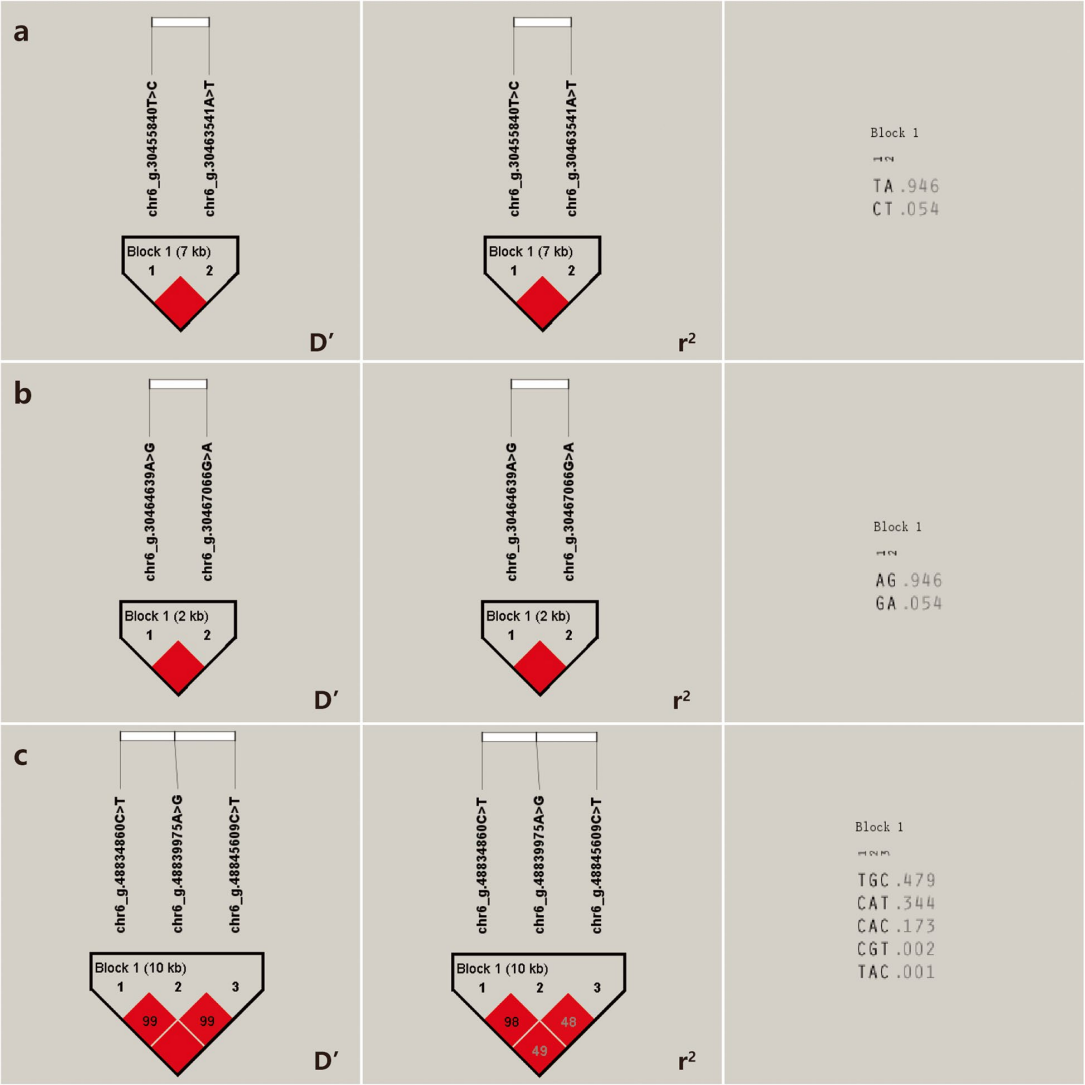
Results of linkage disequilibrium analysis and haplotype block of significantly associated SNPs of cashmere diameter in IMCGs. **a** D' value, *r*^2^ value and haplotype frequency of block formed by 2 SNPs in the *PDLIM5* gene. **b** D' value, *r*^2^ value and haplotype frequency of block formed by other 2 SNPs in the *PDLIM5* gene. **c** D' value, *r*^2^ value and haplotype frequency of block formed by 3 SNPs

**Fig. 9:**
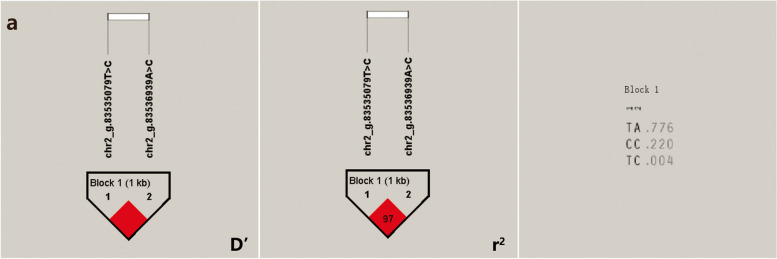
Results of linkage disequilibrium analysis and haplotype block of significantly associated SNPs of cashmere thickness in IMCGs. **a** D' value, *r*^2^ value and haplotype frequency of block formed by 2 SNPs in the *GTDC1* gene

The 7 SNPs significantly associated with the Inner Mongolia cashmere goats cashmere diameter trait constituted 3 Blocks (Fig. 8), respectively. Complete linkage (D' = 1) between chr6_g.30455840 T > C and chr6_g.30463541A > T, and complete linkage between chr6_g.30464639A > G and chr6_g.30467066G > A of *PDLIM5* gene; There was a strong linkage disequilibrium (D' > 0.8 and r^2^ > 0.33) between chr6_g. 48834860C > T and chr6_g. 48839975A > G, chr6_g. 48845609C > T. The 2 SNPs significantly associated with the cashmere thickness trait in Inner Mongolia cashmere goats constituted 1 Block (Fig. 9), with a complete linkage (D' = 1) between chr2_g.83535079 T > C and chr2_g.83536939A > C of *GTDC1* gene.

**Fig. 10:**
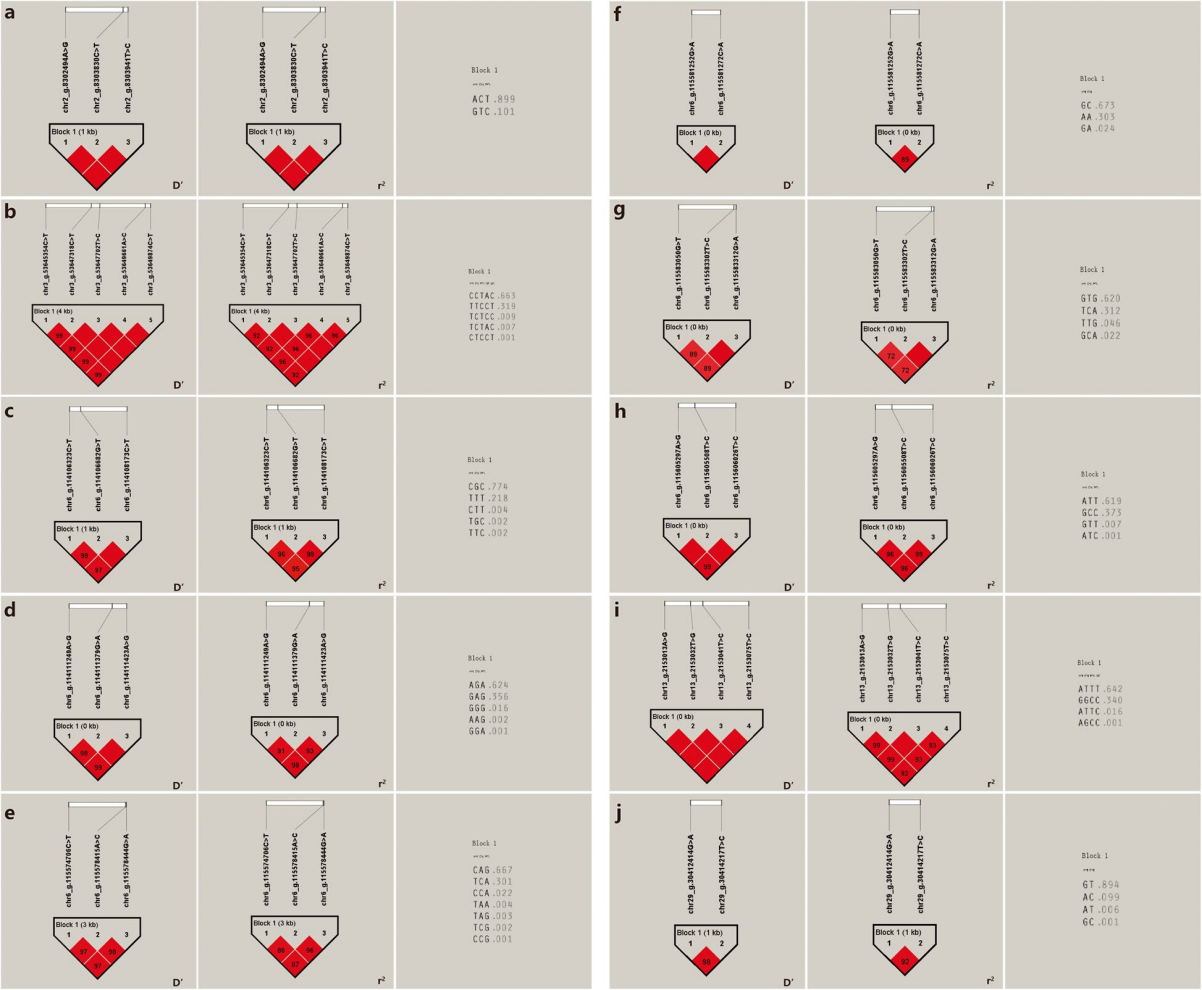
Results of linkage disequilibrium analysis and haplotype block of significantly associated SNPs of fleece length in IMCGs. **a** D' value, *r*^2^ value and haplotype frequency of block formed by 3 SNPs in the *RHCE* gene. **b** D' value, *r*^2^ value and haplotype frequency of block formed by 5 SNPs in the *PIGK* gene. **c** D' value, *r*^2^ value and haplotype frequency of block formed by 3 SNPs in the *SORCS2* gene. **d** D' value, *r*^2^ value and haplotype frequency of block formed by other 3 SNPs in the *SORCS2* gene. **e** D' value, *r*^2^ value and haplotype frequency of block formed by 3 SNPs in the *RGS12* gene. **f** D' value, *r*^2^ value and haplotype frequency of block formed by 2 SNPs in the *RGS12* gene. **g** D' value, *r*^2^ value and haplotype frequency of block formed by 2 SNPs in the *RGS12* gene. **h** D' value, *r*^2^ value and haplotype frequency of block formed by 3 SNPs in the *RGS12* gene. **i** D' value, *r*^2^ value and haplotype frequency of block formed by 4 SNPs in the *PLCB4* gene. **j** D' value, *r*^2^ value and haplotype frequency of block formed by 2 SNPs

The 31 SNPs significantly associated with fleece length in Inner Mongolia cashmere goats constituted 10 Blocks, respectively (Fig. 10), and chr2_g.8302494A > G and chr2_g.8303830C > T and chr2_g.8303941 T > C of the *RHCE* gene were complete linkage (D' = 1) to each other SNPs; There was a strong linkage disequilibrium (D' > 0.8 and r^2^ > 0.33) between chr3_g.53645354C > T and chr3_g.53647318C > T, chr3_g.53647702 T > C, chr3_g.53649661A > C, and chr3_g.53649874C > T of the *PIGK* gene, and complete linkage (D' = 1) between chr3_g.53647318C > T, chr3_g.53647702 T > C, chr3_g.53649661A > C, and chr3_g.53649874C > T; Strong linkage disequilibrium (D' > 0.8 and *r*^2^ > 0.33) between chr6_g.114106323C > T and chr6_g.114106682G > T, chr6_g.114108173C > T of the *SORCS2* gene and chr6_g.114106682G > T, chr6_g.114108173C > T are complete linkage (D' = 1) between them; Strong linkage disequilibrium (D' > 0.8 and *r*^2^ > 0.33) between chr6_g.114111249A > G and chr6_g.114111379G > A and chr6_g.114111423A > G of *SORCS2* gene, and strong linkage disequilibrium (D' > 0.8 and *r*^2^ > 0.33) between chr6_g.114111379G > A and chr6_g.114111423A > G are complete linkage (D' = 1). Strong linkage disequilibrium (D' > 0.8 and *r*^2^ > 0.33) exists between chr6_g.115583050G > T and chr6_g.115583302 T > C and chr6_g.115583312G > A of *RGS12* gene,and complete linkage between chr6_g.115583302 T > C and chr6_g.115583312G > A (D' = 1); Complete linkage (D' = 1) between the chr6_g.115605508 T > C of the *RGS12* gene and chr6_g.115605297A > G, chr6_g.115606026 T > C, and there is a strong linkage disequilibrium (D' > 0.8 and *r*^2^ > 0.33) between chr6_g.115605297A > G and chr6_g.115606026 T > C. There was a complete linkage (D' = 1) between chr13_g.2153013A > G and chr13_g.2153032 T > G, chr13_g.2153041 T > C, and chr13_g.2153075 T > C of *PLCB4* gene. There was a strong linkage disequilibrium (D' > 0.8 and *r*^2^ > 0.33) between chr29_g.30412414G > A and chr29_g.30414217 T > C.

Calculation and analysis of haplotype types and their frequencies (Table 6) showed that the 4 blocks composed of SNPs significantly associated with Inner Mongolia cashmere goats cashmere production actually formed 2, 2, 5 and 4 haplotypes, while the theoretical number of haplotypes were 2^2^ (4), 2^2^ (4), 2^9^ (512) and 2^2^ (4), and the haplotypes of the 4 blocks were named as A1 ~ A2, B1 ~ B2, C1 ~ C5 and D1 ~ D4, and the haplotype frequencies are shown in Table 6. The 3 blocks composed of SNPs that were significantly associated with cashmere diameter in the Inner Mongolia cashmere goat population formed 2, 2 and 5 haplotypes, respectively, whereas the theoretical number of haplotypes was 2^2^ (4), 2^2^ (4) and 2^3^ (8). The haplotypes of the three blocks were named E1 ~ E2, F1 ~ F2 and G1 ~ G5, and the haplotype frequencies are shown in Table 6; 1 block composed of SNPs that were significantly associated with cashmere thickness of Inner Mongolia cashmere goats actually formed 3 haplotypes, whereas the theoretical number of haplotypes was 2^2^ (4), and the haplotypes of this block were named H1 ~ H3, and the haplotype frequencies are shown in Table 6; The 10 SNPs significantly associated with the fleece length of Inner Mongolia cashmere goats actually formed 2, 5, 5, 7, 3, 4, 4, 4, and 2 haplotypes, with theoretical haplotypes of 2^3^ (8), 2^5^ (32), 2^3^ (8), 2^3^ (8), 2^3^ (8), 2^2^ (4), 2^3^ (8), 2^3^ (8), 2^4^ (16), and 2^2^ (4), respectively. The haplotypes of the 10 blocks were named I1 ~ I2, J1 ~ J5, K1 ~ K5, L1 ~ L5, M1 ~ M7, N1 ~ N3, O1 ~ O4, P1 ~ P4, Q1 ~ Q4, and R1 ~ R4, respectively, the haplotype frequencies are shown in Table 6.

**Table 6:** Haplotype combinations and frequencies associated of cashmere traits in IMCGs

Traits	Number of SNP	Tag	Haplotype	Frequency
Cashmere yield	SNP4, SNP5	A1	CG	0.114
		A2	TA	0.886
	SNP6, SNP7	B1	AA	0.114
		B2	GG	0.886
	SNP13, SNP14, SNP15, SNP16, SNP17, SNP18, SNP19, SNP20, SNP21	C1	CGGATACTA	0.004
		C2	CTGGCCTTG	0.922
		C3	CTGGTATTG	0.005
		C4	GGAATACCA	0.068
		C5	GTGGCCTTG	0.001
	SNP27, SNP28	D1	AA	0.054
		D2	AG	0.002
		D3	GA	0.001
		D4	GG	0.942
Cashmere diameter	SNP34, SNP35	E1	CT	0.054
		E2	TA	0.946
	SNP36, SNP37	F1	AG	0.946
		F2	GA	0.054
	SNP38, SNP39, SNP41	G1	CAC	0.173
		G2	CAT	0.344
		G3	CGT	0.002
		G4	TAC	0.001
		G5	TGC	0.479
Cashmere thickness	SNP67, SNP68	H1	CC	0.22
		H2	TA	0.776
		H3	TC	0.004
Fleece length	SNP87, SNP88, SNP89	I1	ACT	0.899
		I2	GTC	0.101
	SNP92, SNP94, SNP95, SNP96, SNP97	J1	CCTAC	0.663
		J2	CTCCT	0.001
		J3	TCTAC	0.007
		J4	TCTCC	0.009
		J5	TTCCT	0.319
	SNP108, SNP109, SNP110	K1	CGC	0.774
		K2	CTT	0.004
		K3	TGC	0.002
		K4	TTC	0.002
		K5	TTT	0.218
	SNP112, SNP113, SNP114	L1	AAG	0.002
		L2	AGA	0.624
		L3	GAG	0.356
		L4	GGA	0.001
		L5	GGG	0.016
	SNP116, SNP119, SNP120	M1	CAG	0.667
		M2	CCA	0.022
		M3	CCG	0.001
		M4	TAA	0.004
		M5	TAG	0.003
		M6	TCA	0.301
		M7	TCG	0.002
	SNP121, SNP122	N1	AA	0.303
		N2	GA	0.024
		N3	GC	0.673
	SNP123, SNP124, SNP125	O1	GCA	0.022
		O2	GTG	0.62
		O3	TCA	0.312
		O4	TTG	0.046
	SNP130, SNP131, SNP132	P1	ATC	0.001
		P2	ATT	0.619
		P3	GCC	0.373
		P4	GTT	0.007
	SNP134, SNP135, SNP136, SNP137	Q1	AGCC	0.001
		Q2	ATTC	0.016
		Q3	ATTT	0.642
		Q4	GGCC	0.34
	SNP150, SNP151	R1	AC	0.099
		R2	AT	0.006
		R3	GC	0.001
		R4	GT	0.894

### Association analysis of haplotype combinations with cashmere traits

**Fig. 11:**
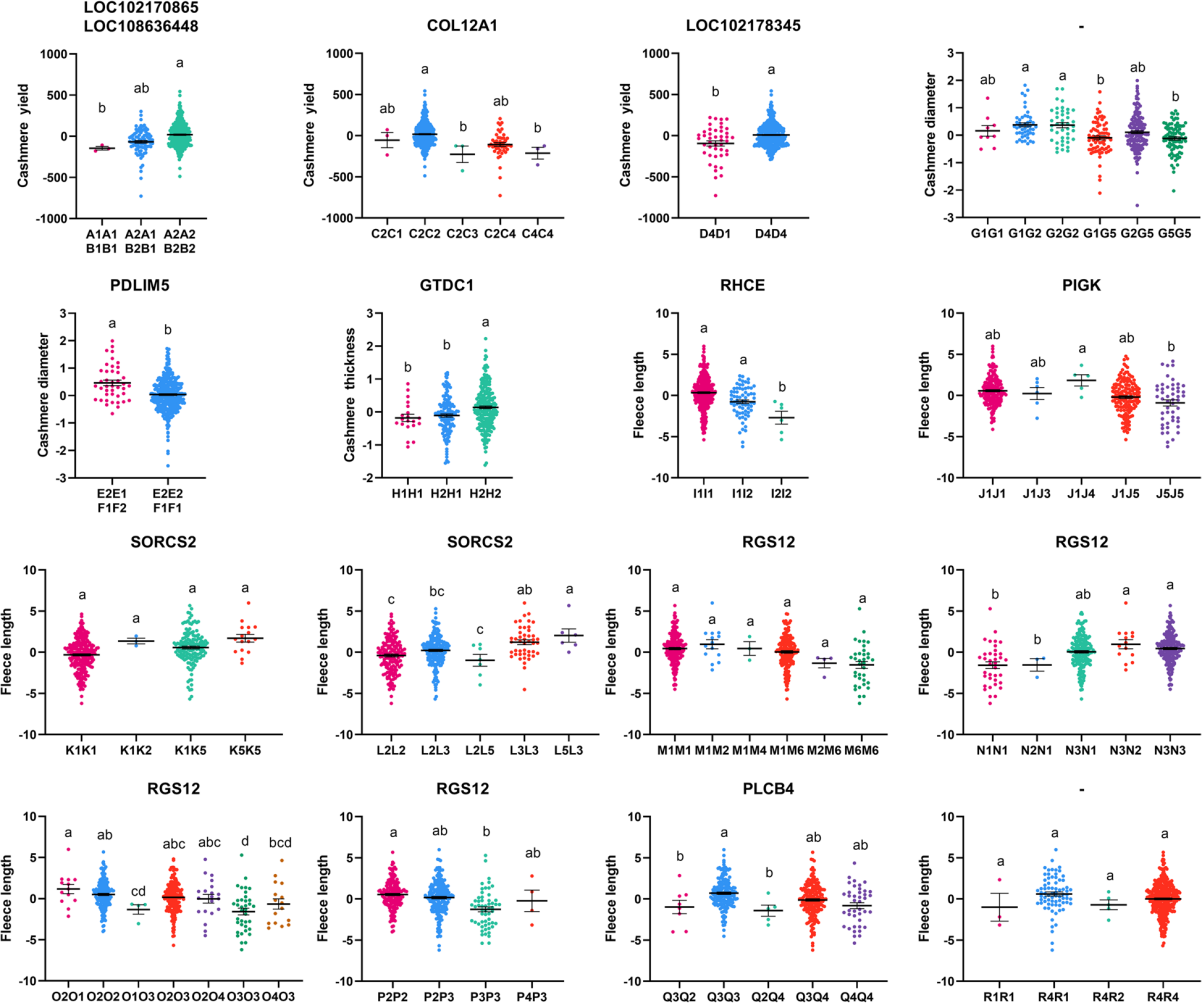
Association analysis between haplotype combinations and phenotypes of cashmere traits in IMCGs. Different letters indicate significant difference, and the same letters indicate insignificant difference

The association analysis of haplotype combinations associated with cashmere traits and cashmere trait phenotypes in Inner Mongolia cashmere goats is shown in Fig. 11 and Table S6. Haplotype combinations with the number of individuals less than 3 were not involved in the multiple comparisons, and among the haplotype combinations constructed by the SNPs that were significantly associated with the cashmere traits, we obtained 3, 3, 5, and 2 haplotype combinations associated with the cashmere yield trait, 2, 2, and 6 haplotype combinations associated with cashmere diameter trait, 3 haplotype combinations associated with cashmere thickness traits, and 3, 5, 4, 5, 6, 5, 7, 4, 5, and 4 haplotype combinations associated with fleece length trait, respectively.

The cashmere yield of the A2A2 haplotype combination of the *LOC102170865* gene was significantly higher than that of the A1A1 haplotype combination (*P* < 0.05), the B2B2 haplotype combination of the *LOC108636448* gene was significantly higher than that of the B1B1 haplotype combination (*P* < 0.05), the C2C2 haplotype combination of the *COL12A1* gene was significantly higher than that of the C2C3 and C4C4 haplotype combination (*P* < 0.05), and the D4D4 haplotype combination of the *LOC102178345* gene was significantly higher than that of the D4D1 haplotype combination (*P* < 0.05). The cashmere diameter of the E2E2 haplotype combination of *PDLIM5* gene is significantly better than that of the E2E1 haplotype combination (*P* < 0.05), the cashmere diameter of the F1F1 haplotype combination of *PDLIM5* gene is significantly better than that of the F1F2 haplotype combination (*P* < 0.05), and the cashmere diameter of the G5G5 and G1G5 haplotype combination is significantly better than that of the G2G2 and G1G2 haplotype combination (*P* < 0.05). The cashmere thickness of the H2H2 haplotype combination of the *GTDC1* gene was significantly higher than that of the H2H1 and H1H1 haplotype combinations (*P* < 0.05). The fleece length of the I1I1 and I1I2 haplotype combinations of the *RHCE* gene was significantly higher than that of the I2I2 combination (*P* < 0.05), the hair length of J1J4 of the *PIGK* gene was significantly higher than that of J5J5 combination (*P* < 0.05), the fleece length of the L5L3 haplotype combination of the *SORCS2* gene was significantly higher than that of L2L3, L2L2, and L2L5 combination (*P* < 0.05), the N3N2 and N3N3 haplotype combinations of the *RGS12* gene were significantly higher than those of N2N1 and N1N1 combination (*P* < 0.05), and the O2O1 haplotype combination of the *RGS12* gene was significantly higher than that of O2O3 The haplotype combination of O3O3 and O4O3 (*P* < 0.05), the P2P2 haplotype combination of *RGS12* gene was significantly higher than the P3P3 haplotype combination (*P* < 0.05), and the Q3Q3 haplotype combination of *PLCB4* gene was significantly higher than the Q3Q2 and Q2Q4 combinations (*P* < 0.05). There was no significant difference (*P* > 0.05) between haplotype combinations of other genes.

### KASP validated SNPs polymorphism

**Fig. 12:**
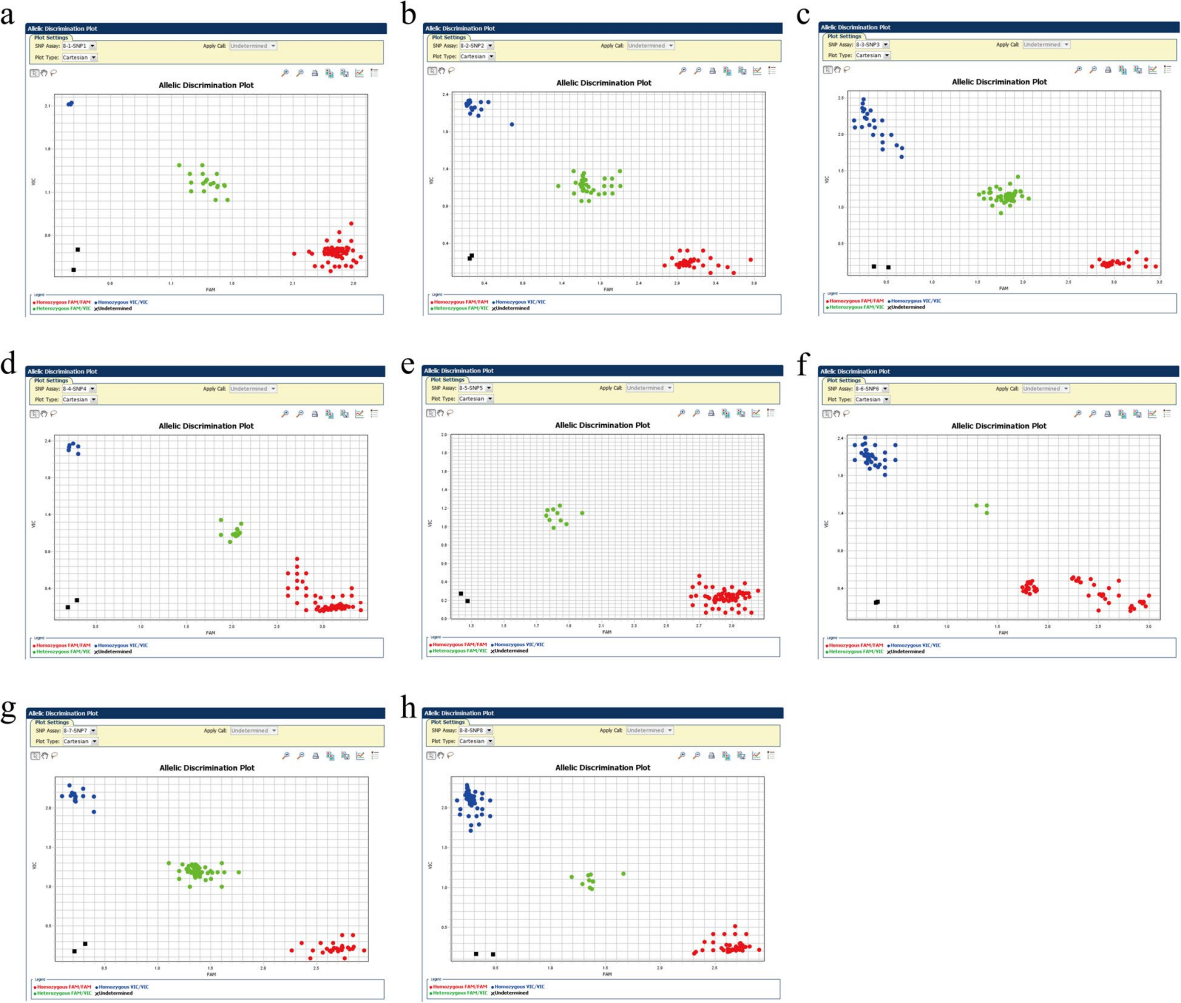
Results of KASP genotyping analysis of 8 SNPs in Inner Mongolia cashmere goats. **a**-**h** represents chr7_g.102631194A > G, chr10_g.82715068 T > C, chr1_g.124483769C > T, chr6_g.30463541A > T, chr24_g.12811352C > T, chr24_g.14180758C > T, chr6_g.114111249A > G, and chr6_g.115606026 T > C, respectively. Genotyped samples marked red are wild type. Genotyped samples marked blue are homozygous mutant type. Genotyped samples marked green are heterozygous mutant type. Genotyped samples marked black are non template contrast

The KASP genotyping results of 8 SNPs in Inner Mongolia cashmere goats were shown in Fig. 12 and Table 7, and all SNPs were successfully genotyped in 96 individuals. Correlation analysis between SNP and cashmere traits showed that the cashmere yield of AA genotype with chr7_g.102631194A > G was significantly increased by 140.75 g compared with AG genotype (*P* = 0.0017). The cashmere yield of the CC genotype with chr10_g.82715068 T > C was significantly increased by 160.77 g compared with the TT genotype (*P* = 0.0165). The cashmere diameter of the TT genotype with chr1_g.124483769C > T was significantly decreased by 2.085 μm compared with the CC genotype (*P* = 0.0306). The cashmere thickness of CC genotype with chr24_g.12811352C > T was significantly increased by 0.881 cm compared with CT genotype (*P* = 0.0389). The fleece length of the GG genotype with chr6_g.114111249A > G was significantly increased by 2.261 cm compared with the AA genotype (*P* = 0.0220). The fleece length of the CC genotype with chr6_g.115606026 T > C was significantly increased by 1.597 cm compared with the TT genotype (*P* = 0.0161). The correlation analysis results of chr6_g.30463541A > T and chr24_g.14180758C > T with cashmere traits were not significant (*P* = 0.1052, *P* = 0.8766) (Table 8).

**Table 7:** Genotyping of 8 SNPs in Inner Mongolia cashmere verified populations

Cashmere traits (Number of samples)	SNP	Genotype	Number	Frequency
Cashmere yield (96)	chr7_g.102631194A > G	AA	74	77.1%
		AG	19	19.8%
		GG	3	3.1%
	chr10_g.82715068 T > C	TT	35	36.5%
		TC	44	45.8%
		CC	17	17.7%
Cashmere diameter (96)	chr1_g.124483769C > T	CC	28	29.2%
		CT	44	45.8%
		TT	24	25.0%
	chr6_g.30463541A > T	AA	78	81.3%
		AT	12	12.5%
		TT	6	6.3%
Cashmere thickness (96)	chr24_g.12811352C > T	CC	86	89.6%
		CT	10	10.4%
		TT	0	0.0%
	chr24_g.14180758C > T	CC	56	58.3%
		CT	3	3.1%
		TT	37	38.5%
Fleece length (96)	chr6_g.114111249A > G	AA	29	30.2%
		AG	53	55.2%
		GG	14	14.6%
	chr6_g.115606026 T > C	TT	37	38.5%
		TC	12	12.5%
		CC	47	49.0%

**Table 8:** Association analysis of verified SNPs with cashmere traits

SNP	Wild type	Heterozygous mutant type	Homozygous mutant type	*P* values	Adjusted *P* values
chr7_g.102631194A > G	606.014 ± 23.600^a^	465.263 ± 28.917^b^	535.000 ± 170.758^ab^	0.0020	0.0017
chr10_g.82715068 T > C	498.057 ± 20.897^b^	605.864 ± 30.150^a^	658.824 ± 65.483^a^	0.0084	0.0165
chr1_g.124483769C > T	16.689 ± 0.534^a^	15.325 ± 0.440^ab^	14.604 ± 0.568^b^	0.0291	0.0306
chr6_g.30463541A > T	15.163 ± 0.325^a^	17.059 ± 0.870^a^	17.438 ± 0.905^a^	0.0286	0.1052
chr24_g.12811352C > T	5.081 ± 0.136^a^	4.200 ± 0.389^b^	-	0.0389	-
chr24_g.14180758C > T	4.955 ± 0.178^a^	5.333 ± 0.882^a^	5.014 ± 0.200^a^	0.8766	-
chr6_g.114111249A > G	11.310 ± 0.587^b^	12.774 ± 0.279^a^	13.571 ± 0.747^a^	0.0110	0.0220
chr6_g.115606026 T > C	11.595 ± 0.365^b^	12.167 ± 0.520^ab^	13.192 ± 0.425^a^	0.0188	0.0161

Note: Different letters indicate significant difference, and the same letters indicate insignificant difference

## Discussion

In this study, the estimated heritability values of cashmere yield, cashmere diameter, and fleece length traits of Inner Mongolia cashmere goats were 0.229, 0.359, and 0.250, respectively, indicating moderate heritability. The estimated heritability of cashmere thickness is 0.053, which is a low heritability trait. In other studies, the heritability of Inner Mongolia cashmere goat cashmere yield was 0.24, 0.30, and 0.34, and the heritability of cashmere diameter was 0.27, 0.28, and 0.32, respectively. The heritability of fleece length was 0.29, 0.31, and 0.32, respectively, all belonging to the medium heritability, which was consistent with the results of this study. For cashmere thickness traits, the results of this study are lower than those of Fenghong Wang, Xuewu Li, and Junyan Bai (0.14, 0.17, and 0.21) [20–22], which may be due to the difference in the number of effective data pieces and estimation methods. Although there are some differences between this study and some other studies, most studies show that cashmere thickness is a low heritability trait.

This study showed that cashmere yield was positively correlated with cashmere diameter, cashmere thickness, and fleece length, indicating that the higher the cashmere yield, the coarser the cashmere diameter, the greater the cashmere thickness, the longer the fleece length. We found that the genetic correlation between cashmere yield and cashmere thickness was as high as 0.835, indicating that the cashmere yield of Inner Mongolia cashmere goats was largely determined by cashmere thickness. In other words, as cashmere thickness increases, cashmere yield increases significantly. The genetic correlation between cashmere yield and cashmere diameter is 0.368, indicating that cashmere grows thicker with the increase in cashmere yield, which is contrary to our breeding goal of "Inner Mongolia cashmere goats producing high yield and good quality cashmere." In the process of breeding, it is difficult to achieve both cashmere yield and cashmere quality, so in practice, we should establish different core groups according to the needs, such as fine cashmere goats and high-yield cashmere goats. The genetic correlation between cashmere thickness and fleece length was 0.475, indicating that cashmere thickness traits could be indirectly increased and improved by fleece length breeding. Cashmere thickness is closely related to cashmere yield. Therefore, cashmere yield can be increased by breeding long fleece cashmere goats, which will lay the foundation for the development of new Inner Mongolia cashmere goats with high yield and high quality cashmere.

Here, we conducted a GWAS work based on Inner Mongolia cashmere goat population. In the GWAS analysis, SNP effect value and significance *P*-value are two different concepts, and it is not the case that the larger the effect value, the more significant it is. In fact, the SNP with small effect values but significant belong to that the SNP have a direct effect on the phenotypic value. However, the effect of the influence is smaller and more stable, which is why they show a highly significant and small effect. For example, chr9_g.1627149C > T and chr9_g.1627193C > A on chromosome 9, which affect the trait of cashmere yield, are significant but have small effect values. the SNP with large effect values but insignificant may be that the SNPs are easily affected by the environment, or the SNPs themselves may be insignificant. Among these 151 SNP, a considerable number of SNP explained more than 2% of the phenotypic variation, such as in the results of cashmere diameter trait, the SNP that explained the largest phenotypic variation was chr7_g.10569445C > T, and the phenotypic variation that this SNP could explain was 3.97%. Although these SNPs may not be QTNs (quantitative trait nucleotides) directly affecting the target traits, their high rate of explained phenotypic variation suggests that these SNPs can be used in marker-assisted selection as well as genome-wide selection to improve selection for cashmere traits in Inner Mongolia cashmere goats.

In this study, we performed GWAS and identified a large number of candidate genes associated with hair follicle growth and development. *NOTCH* genes are expressed in epithelial cells, epidermal keratinocytes, follicular cells and sebaceous glands. Significant SNPs associated with cashmere yield: high expression of the *Notch3* gene annotated to chr7_g.100291226 T > C promotes fibroblast hyperactivation [23]. According to clusterProfiler enrichment analysis, *Notch3* is involved in the Notch signaling pathway for intercellular signaling, which is essential for normal embryonic development in mammals. Significant SNPs associated with cashmere yield: chr6_g.3398555C > T, the annotated *CCNA2* gene belongs to a highly conserved family of cell cycle proteins that binds to and activates *CDC2* or *CDK2* kinases, thereby promoting the cell cycle G1/S and G2/M transitions [24].

In addition, it has been shown that 2 SNPs on *CCNA2* have potential effects on hair density in otter rabbits [25]. *CCNA2* can affect the cell cycle of hair papilla cells and hair follicle stem cells [26, 27]. Significant SNPs associated with cashmere diameter: chr6_g.30455840 T > C annotated to *BMPR1B* showed a trend of cycle-specific differential expression in porcine embryonic skin tissues [28], also enriched for the TGF-beta signaling pathway that regulates cellular function. Hair follicle stem cells are key to promoting hair follicle growth and homeostasis, and have a significant hair cycling regenerative capacity, and exhibit fate plasticity during skin trauma healing. It has been shown that *LGR5* is a marker of homeostasis and development of hair follicle stem cells across species [29]. Significant SNP associated with fleece length: chr26_g.7282942G > T annotated to *CTBP2* is stably expressed in goat skin tissues [30]. chr21_g.65871544A > G annotated *PPP2R5C* is essential in mammalian growth and development, and it has been shown that *PPP2R5C* g.65977743C > T is significantly associated with the number of third lactation litters of the Yunshang black goats [31]. chr2_g.8298760A > T annotated *STMN1* promotes the proliferative process of hair follicle cells in mice [32]. *STMN1* was also enriched in the MAPK signaling pathway involved in cell proliferation, differentiation and migration, implying that the gene could regulate cell cycle process. When screening candidate genes affecting the four traits, we found that SNPs significantly associated with cashmere yield and fleece length were co-annotated to the *HMX1*, *ADRA2C*, *AFAP1* and *ABLIM2* genes, suggesting that these four genes can regulate both cashmere yield and fleece length, and that by focusing on these genes in the selection and breeding process, we can select goat individuals with high cashmere yield and longer fleece. Moreover, as fleece length increases, so do cashmere yield and body weight [33]. Selection of long fleece individuals to be retained in the breed can accelerate the genetic progression of cashmere yield and body weight in the cashmere goat, and realize the indirect selection of fleece for cashmere yield and body weight.

Haplotype information is widely used in linkage analysis, correlation studies, population genetics, etc. [34]. Haplotype analysis provides more comprehensive information, and more accurate statistical results can be obtained using haplotypes than single marker analysis [35]. In the genetic process, SNPs tend to be inherited as a whole to the offspring, if the density of significant SNPs obtained through GWAS is low, the range of localization intervals will increase at this time, which will cause difficulties in the subsequent search for candidate genes, and the construction of haplotypes is undoubtedly the best solution. Through statistical methods, a class of SNPs with correlations on chromosomes constitutes a collection, which is used to reflect the genetic effects of these SNPs. The advantage of the single-SNP GWAS approach is that errors in mapping order have no effect on the SNPs, but may lead to erroneous inference of haplotype alleles. In addition, the haplotype GWAS method is based on a chi-square distribution, which has more degrees of freedom than a single SNP, so for the same *p*-value, the likelihood ratio needs to be higher to reach the significance threshold [36]. However, haplotype GWAS seems to be more advantageous for detecting complex signals because of the diversity of combinations of alleles in haplotypes. For example, there are more than two alleles in a causal mutation associated with a trait or two possible mutations in the same gene. Martin et al. [37] in exploring novel loci associated with coat color traits in Saanen goats found that both the single SNP GWAS and haplotype GWAS methods detected the highest signals for the coat color trait, whereas the smaller signals detected by the two methods did not coincide, and such a results do not exclude the possibility of false positives, and in the process of performing haplotype GWAS, factors such as family structure are also likely to have an impact on the accuracy of haplotype construction. Zhu et al. [38] performed GWAS analysis of six erythrocyte traits in 498 Alpine Merino sheep based on the single SNP and haplotype methods, and the significant candidate genes obtained by the single SNP GWAS and haplotype GWAS methods were taken to intersections and screened three significant genes, *DHCR24*, *PLCB1* and *SPATA9*. In addition, *FLI1*, an important gene related to hematopoiesis, was also obtained by haplotype GWAS method. This suggests that we can make the results more reliable by utilizing overlapping markers detected by two different methods, while non-overlapping markers may represent new valuable associations detected by different methods. In the present study, haplotypes A2A2, B2B2, C2C2, and D4D4 were associated with elevated cashmere yield, haplotypes E2E2, F1F1, G5G5, and G1G5 were associated with reduced cashmere fineness, haplotype H2H2 was associated with elevated cashmere thickness, and haplotypes I1I1, I1I2, J1J4, L5L3, N3N2, and N3N3, O2O1, P2P2, and Q3Q3 were associated with increased cashmere length. Multiple alleles in the haplotype are co-inherited on the same chromosome and collectively influence cashmere traits. A series of SNPs in haplotypes that affect the cashmere traits of Inner Mongolia cashmere goats can lay a theoretical foundation for molecular marker-assisted selection of cashmere traits of Inner Mongolia cashmere goats.

We verified that 8 newly discovered SNPs, chr7_g.102631194A > G, chr10_g.82715068 T > C, chr1_g.124483769C > T, chr24_g.12811352C > T, chr6_g.114111249A > G, and chr6_g.115606026 T > C were significantly genotyped in verified populations (*P* < 0.05). The correlation analysis results of chr6_g.30463541A > T and chr24_g.14180758C > T with cashmere traits were not significant, possibly because the *P* value after Bonferroni correction could not reach the significance level due to the stricter standard [39]. It is also possible that the sample size of the verification group is small, sample sizes can provide imprecise or incorrect estimates of the magnitude of the observed effects [40]. However, the limitation of this study is that only some Inner Mongolia cashmere goats were investigated. Therefore, it is unclear whether these results can be determined and generalized to other goat breeds. In future studies, goats from different pastures, more goat samples and goat breeds will be investigated to further confirm the results of this study and to discover more valuable new variants.

## Conclusions

Using the sum of estimated breeding values and residuals as corrected phenotypic values, we detected 151 genome-wide SNPs significantly associated with four cashmere traits in the Inner Mongolia cashmere goat population, and constructed 21 haplotype blocks and 68 haplotype combinations. Among them, chr7_g.102631194A > G, chr10_g.82715068 T > C, chr1_g.124483769C > T, chr24_g.12811352C > T, chr6_g.114111249A > G, and chr6_g.115606026 T > C were significantly genotyped in verified populations (*P* < 0.05). These SNPs can be used as candidate sites for molecular marker-assisted selection of cashmere traits in Inner Mongolia cashmere goats. In the subsequent research, it is necessary to further expand the population size and sequencing depth, and actively carry out functional verification tests, so that these new candidate genes or haplotypes can be more accurately applied to the genetic improvement of Inner Mongolia cashmere goat populations.

### Supplementary Information

Supplementary Material 1.

Supplementary Material 2.

Supplementary Material 3.

Supplementary Material 4.

Supplementary Material 5.

Supplementary Material 6.

Supplementary Material 7.

## Data Availability

The data-sets generated during and analyzed during the current study are available in the article. The data that support the findings of this study are available from College of Animal Science, Inner Mongolia Agricultural University, Hohhot 010018, China (Whole genome re-sequence data). Restrictions apply to the availability of these data, which were used under license for this study. Data are available from the authors with the permission of Inner Mongolia Agricultural University.

## Abbreviations

CY: Cashmere yield
CD: Cashmere diameter
CT: Cashmere thickness
FL: Fleece length
GWAS: Genome-wide associated studies
SNPs: Single nucleotide polymorphisms
QTNs: Quantitative trait nucleotides
LD: Linkage disequilibrium
IMCGs: Inner Mongolia cashmere goats
HWE: Hardy-Weinberg equilibrium test
Ho: Homozygosity
He: Heterozygosity
PIC: Polymorphism information content
Ne: Effective allele numbers
MAF: Minor allele frequency
IBS: Idengtical by state
GO: Gene Ontology
KEGG: Kyoto encyclopedia of genes and genomes

## References

[1] Zhang L, Wang F, Gao G, Yan X, Liu H, Liu Z, Wang Z, He L, Lv Q, Wang Z (2021). Genome-wide association study of body weight traits in Inner Mongolia cashmere goats. Front Vet Sci.

[2] Wang FH, Zhang L, Gong G, Yan XC, Zhang LT, Zhang FT, Liu HF, Lv Q, Wang ZY, Wang RJ (2021). Genome-wide association study of fleece traits in Inner Mongolia cashmere goats. Anim Genet.

[3] Chen S, Zhou Y, Chen Y, Gu J (2018). fastp: an ultra-fast all-in-one FASTQ preprocessor. Bioinformatics (Oxford, England).

[4] Li H, Durbin R (2009). Fast and accurate short read alignment with Burrows-Wheeler transform. Bioinformatics (Oxford, England).

[5] Li H, Handsaker B, Wysoker A, Fennell T, Ruan J, Homer N, Marth G, Abecasis G, Durbin R (2009). The sequence alignment/map format and SAMtools. Bioinformatics (Oxford, England).

[6] McKenna A, Hanna M, Banks E, Sivachenko A, Cibulskis K, Kernytsky A, Garimella K, Altshuler D, Gabriel S, Daly M (2010). The genome analysis toolkit: a MapReduce framework for analyzing next-generation DNA sequencing data. Genome Res.

[7] Wang K, Li M, Hakonarson H (2010). ANNOVAR: functional annotation of genetic variants from high-throughput sequencing data. Nucleic Acids Res.

[8] Purcell S, Neale B, Todd-Brown K, Thomas L, Ferreira MA, Bender D, Maller J, Sklar P, de Bakker PI, Daly MJ (2007). PLINK: a tool set for whole-genome association and population-based linkage analyses. Am J Hum Genet.

[9] Gondro C, Porto-Neto LR, Lee SH (2013). R for genome-wide association studies. Methods Mol Biol (Clifton, NJ).

[10] Zhang H, Liu A, Li X, Xu W, Shi R, Luo H, Su G, Dong G, Guo G, Wang Y (2019). Genetic analysis of skinfold thickness and its association with body condition score and milk production traits in Chinese Holstein population. J Dairy Sci.

[11] Jiang L, Zheng Z, Qi T, Kemper KE, Wray NR, Visscher PM, Yang J (2019). A resource-efficient tool for mixed model association analysis of large-scale data. Nat Genet.

[12] Quinlan AR, Hall IM (2010). BEDTools: a flexible suite of utilities for comparing genomic features. Bioinformatics (Oxford, England).

[13] Wu T, Hu E, Xu S, Chen M, Guo P, Dai Z, Feng T, Zhou L, Tang W, Zhan L (2021). clusterProfiler 4.0: a universal enrichment tool for interpreting omics data. Innovation (Cambridge (Mass)).

[14] Dong SS, He WM, Ji JJ, Zhang C, Guo Y, Yang TL (2021). LDBlockShow: a fast and convenient tool for visualizing linkage disequilibrium and haplotype blocks based on variant call format files. Brief Bioinform.

[15] Barrett JC (2009). Haploview: visualization and analysis of SNP genotype data. Cold Spring Harb Protoc.

[16] Moser EB, Saxton AM, Geaghan JP (1988). Biological applications of the SAS system: an overview. Comput Appl Biosci.

[17] Silió L, Rodríguez MC, Fernández A, Barragán C, Benítez R, Óvilo C, Fernández AI (2013). Measuring inbreeding and inbreeding depression on pig growth from pedigree or SNP-derived metrics. J Anim Breed Genet = Zeitschrift fur Tierzuchtung und Zuchtungsbiologie.

[18] Cai C, Zhang X, Zhang W, Yang Y, Gao P, Gao X, Li B, Cao G (2021). Evaluation of genetic structure in Mashen pigs conserved population based on SNP chip. Acta Vet Zootechnica Sini.

[19] Long JR, Zhao LJ, Liu PY, Lu Y, Dvornyk V, Shen H, Liu YJ, Zhang YY, Xiong DH, Xiao P (2004). Patterns of linkage disequilibrium and haplotype distribution in disease candidate genes. BMC Genet.

[20] Bai J. Genetic evaluation and genetic parameter estimation in Inner Mongolia cashmere goats using animal model BLUP and DFREML. Master thesis. Inner Mongolia Agricultural University, Department of Animal Genetics, Breeding and Reproduction; 2002.

[21] Li X, Wang R, Wang Z, Na Q, Li H, Wang Z, Su R, Zhang Y, Li J, Liu S (2017). Study on the estimation of genetic parameters and genetic progress for fleece traits of Inner Mongolian cashmere goats. Heilongjiang Anim Sci Vet Med..

[22] Wang F. Design of goat SNP chip with applications in genome-wide association study and genomic selection ofimportant economie traits in Inner Mongolia cashmere goat. PhD thesis. Inner Mongolia Agricultural University, Department of Animal Genetics, Breeding and Reproduction; 2021.

[23] Zmorzyński S, Styk W, Filip AA, Krasowska D (2019). The significance of NOTCH pathway in the development of fibrosis in systemic sclerosis. Ann Dermatol.

[24] Jeffrey PD, Russo AA, Polyak K, Gibbs E, Hurwitz J, Massagué J, Pavletich NP (1995). Mechanism of CDK activation revealed by the structure of a cyclinA-CDK2 complex. Nature.

[25] Chen SJ, Liu T, Liu YJ, Dong B, Huang YT, Gu ZL (2011). Identification of single nucleotide polymorphisms in the CCNA2 gene and its association with wool density in rex rabbits. Genet Mol Res : GMR.

[26] Kim J, Shin JY, Choi YH, Jang M, Nam YJ, Lee SY, Jeon J, Jin MH, Lee S (2019). Hair growth promoting effect of Hottuynia cordata extract in cultured human hair follicle dermal papilla cells. Biol Pharm Bull.

[27] Ge M, Liu C, Li L, Lan M, Yu Y, Gu L, Su Y, Zhang K, Zhang Y, Wang T (2019). miR-29a/b1 inhibits hair follicle stem cell lineage progression by spatiotemporally suppressing WNT and BMP signaling. Cell Rep.

[28] Jiang Y, Liu H, Zou Q, Li S, Ding X (2022). miR-29a-5p inhibits prenatal hair placode formation through targeting EDAR by ceRNA regulatory network. Front Cell Dev Bio.

[29] Polkoff KM, Gupta NK, Green AJ, Murphy Y, Chung J, Gleason KL, Simpson SG, Walker DM, Collins B, Piedrahita JA (2022). LGR5 is a conserved marker of hair follicle stem cells in multiple species and is present early and throughout follicle morphogenesis. Sci Rep.

[30] Zhang J, Deng C, Li J, Zhao Y (2020). Transcriptome-based selection and validation of optimal house-keeping genes for skin research in goats (Capra hircus). BMC Genomics.

[31] Wang P, Li W, Liu Z, He X, Lan R, Liu Y, Chu M (2022). Analysis of the association of two SNPs in the promoter regions of the PPP2R5C and SLC39A5 genes with litter size in Yunshang black goats. Animals.

[32] Bichsel KJ, Hammiller B, Trempus CS, Li Y, Hansen LA (2016). The epidermal growth factor receptor decreases Stathmin 1 and triggers catagen entry in the mouse. Exp Dermatol.

[33] Li X, Liu Y, Wang R, Wang Z, Na Q, Li H, Wang Z, Xv B, Su R, Zhang Y (2018). Genetic parameter estimation of cashmere yield and body weight at different staple types of Inner Mongolian cashmere goats. Sci Agric Sin.

[34] Snyder MW, Adey A, Kitzman JO, Shendure J (2015). Haplotype-resolved genome sequencing: experimental methods and applications. Nat Rev Genet.

[35] Sandrim VC, Tanus-Santos JE (2007). Haplotype analysis can provide improved clinical information than single genotype analysis. Thromb Res.

[36] Lorenz AJ, Hamblin MT, Jannink JL (2010). Performance of single nucleotide polymorphisms versus haplotypes for genome-wide association analysis in barley. PLoS One.

[37] Martin PM, Palhière I, Ricard A, Tosser-Klopp G, Rupp R (2016). Genome wide association study identifies new loci associated with undesired coat color phenotypes in Saanen goats. PLoS One.

[38] Zhu S, Guo T, Zhao H, Qiao G, Han M, Liu J, Yuan C, Wang T, Li F, Yue Y (2020). Genome-wide association study using individual single-nucleotide polymorphisms and haplotypes for erythrocyte traits in Alpine Merino sheep. Front Genet.

[39] Curtin F, Schulz P (1998). Multiple correlations and Bonferroni's correction. Biol Psychiatry.

[40] Colhoun HM, McKeigue PM, Davey Smith G (2003). Problems of reporting genetic associations with complex outcomes. Lancet.

