# Supplementary material for: Genome-wide association study for cashmere traits in Inner Mongolia cashmere goat population reveals new candidate genes and haplotypes
Source: BMC Genomics. 2024 Jul 2;25:658. doi: 10.1186/s12864-024-10543-4 (PMC11218406; doi:10.1186/s12864-024-10543-4)
Supplement: Supplementary file 1 — Supplementary Material 1. [file 12864_2024_10543_MOESM1_ESM.docx]

**Supplementary Information**

Genome-wide association study for cashmere traits in Inner Mongolia cashmere goat population reveals new candidate genes and haplotypes

**Rong et al.**


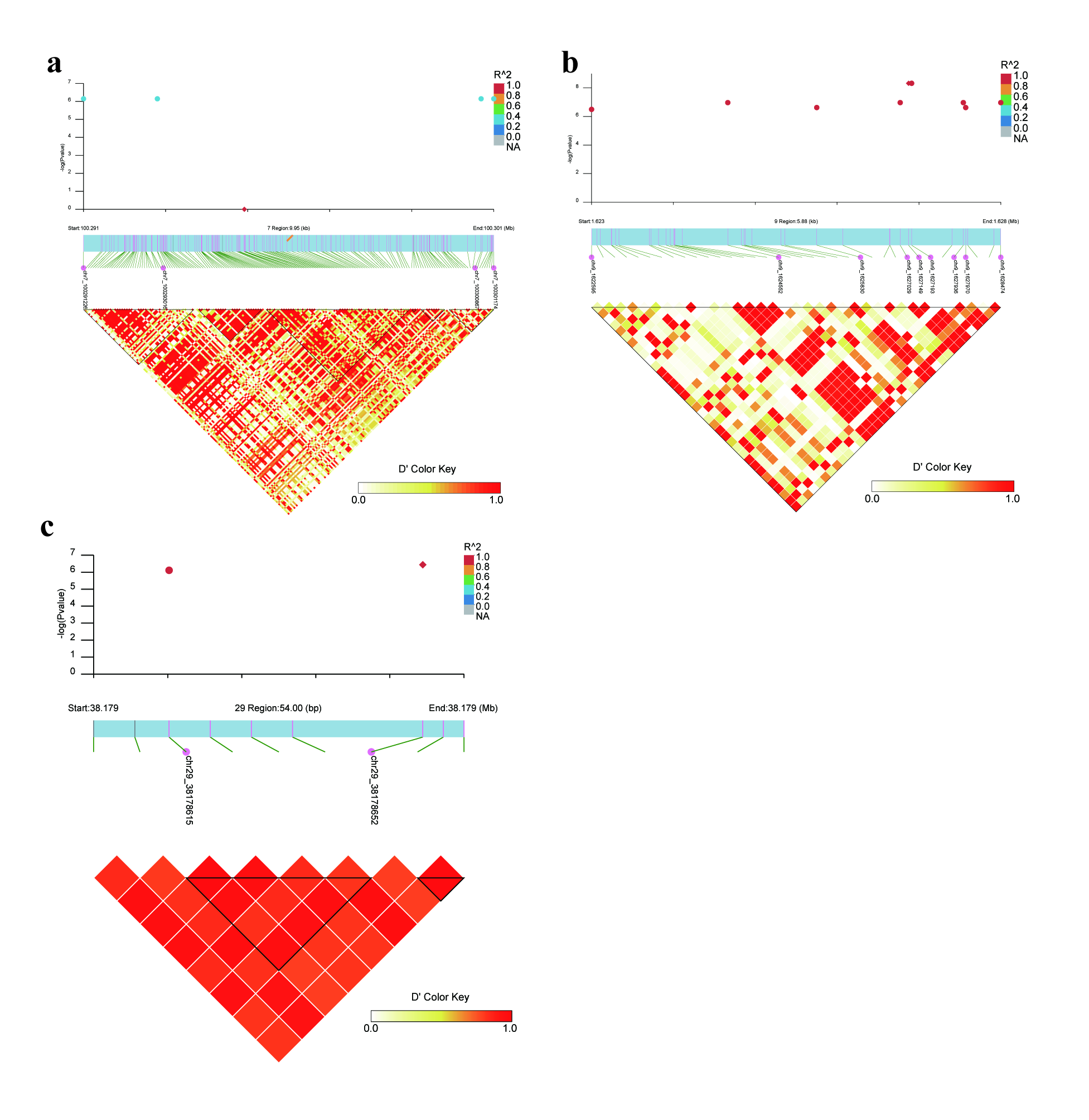


**Supplementary Figure 1.** Construction of haplotype blocks significantly associated with cashmere yield traits. **(a)** represents chr7_g.100291226T>C and chr7_g.100293016A>G are located in 1 Block，chr7_g.100300867G>A and chr7_g.100301174G>A are located in 1 Block; **(b)** represents chr9_g.1622595C>G, chr9_g.1624552T>G, chr9_g.1625830G>A, chr9_g.1627029G>A, chr9_g.1627149C>T, chr9_g.1627193C>A, chr9_g.1627936T>C, chr9_g.1627970T>C, and chr9_g.1628474C>A are in 1 Block; **(c)** represents chr29_g.38178615G>A and chr29_g.38178652G>A are in 1 Block.


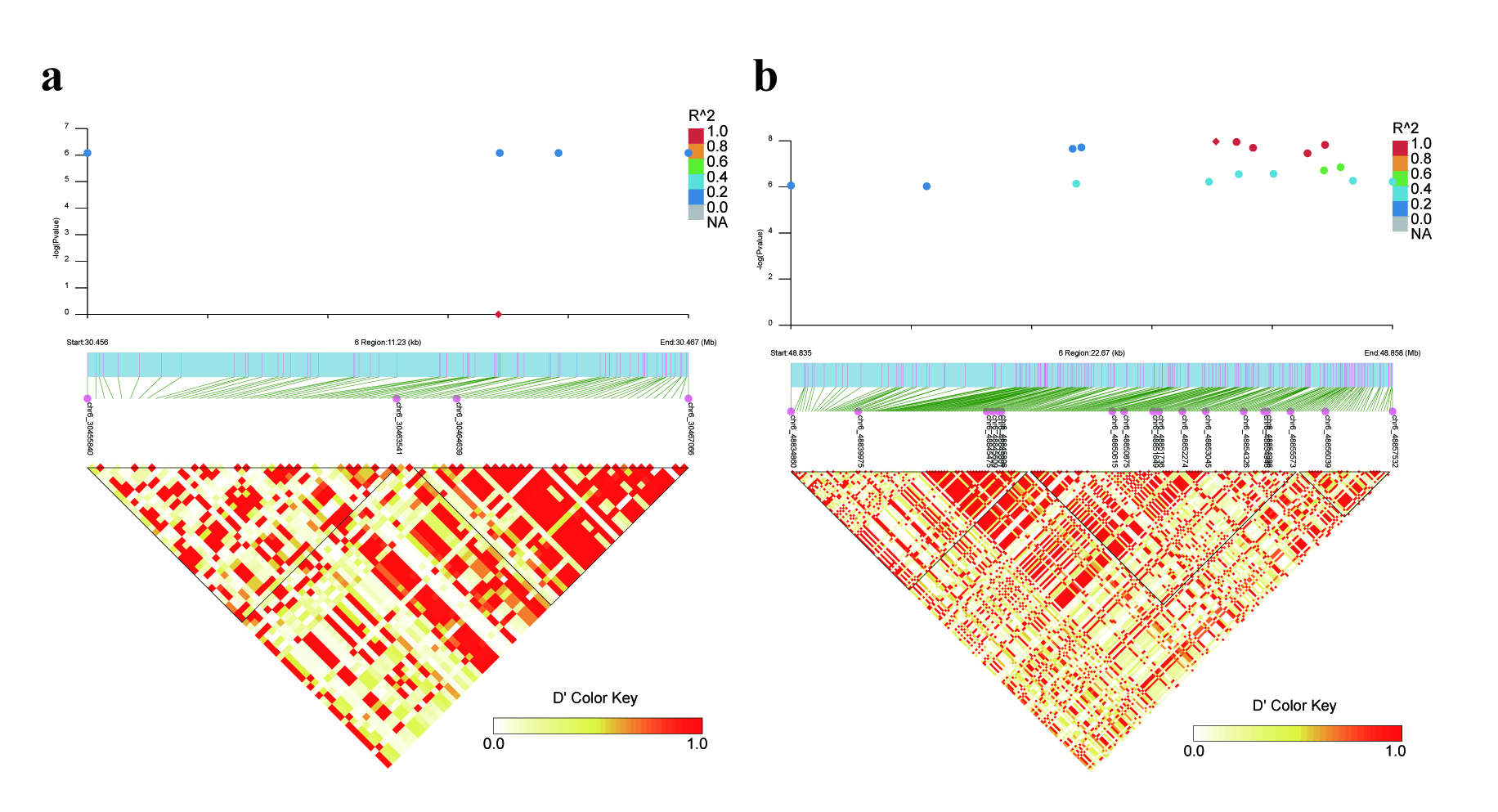


**Supplementary Figure 2.** Construction of haplotype blocks significantly associated with cashmere diameter traits. **(a)** represents chr6_g.30455840T>C and chr6_g.30463541A>T are located in 1 Block; chr6_g.30464639A>G and chr6_g.30467066G>A were located in 1 Block; **(b)** represents chr6_g.48834860C>T, chr6_g.48839975A>G, chr6_g.48845475C>A, chr6_g.48845609C>T, chr6_g.48845801T>A and chr6_g.48845806A>G located in 1 Block; chr6_g.48850615G>A, chr6_g.48850875G>C, chr6_g.48851649T>G, chr6_g.48851736A>G, chr6_g.48852274A>G, chr6_g.48853045 T>C, chr6_g.48854326G>A, chr6_g.48854946G>T, chr6_g.48854986A>G, chr6_g.48855573A>G are located in 1 Block.


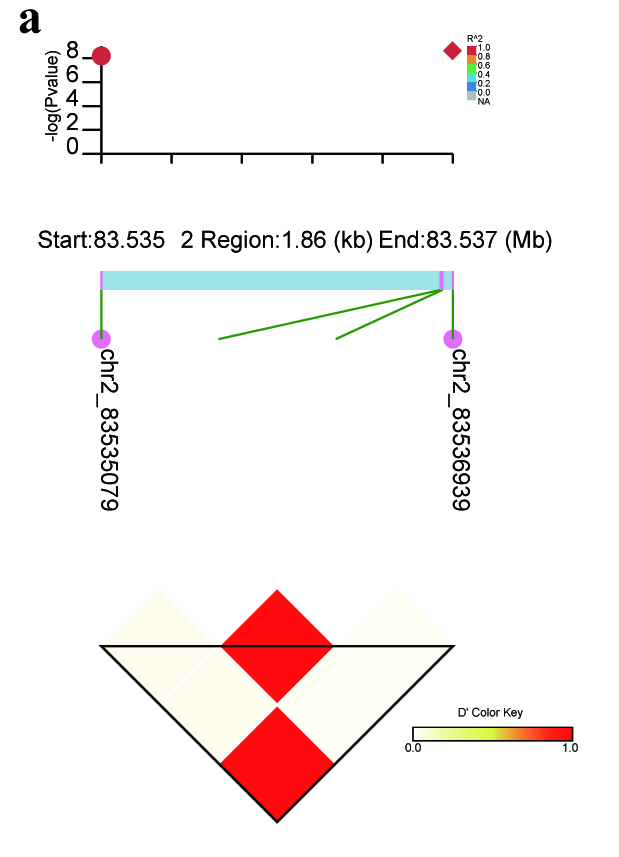


**Supplementary Figure 3.** Construction of haplotype blocks significantly associated with cashmere thickness traits. **(a)** represents chr2_g.83535079T>C and chr2_g.83536939A>C are located in 1 Block.


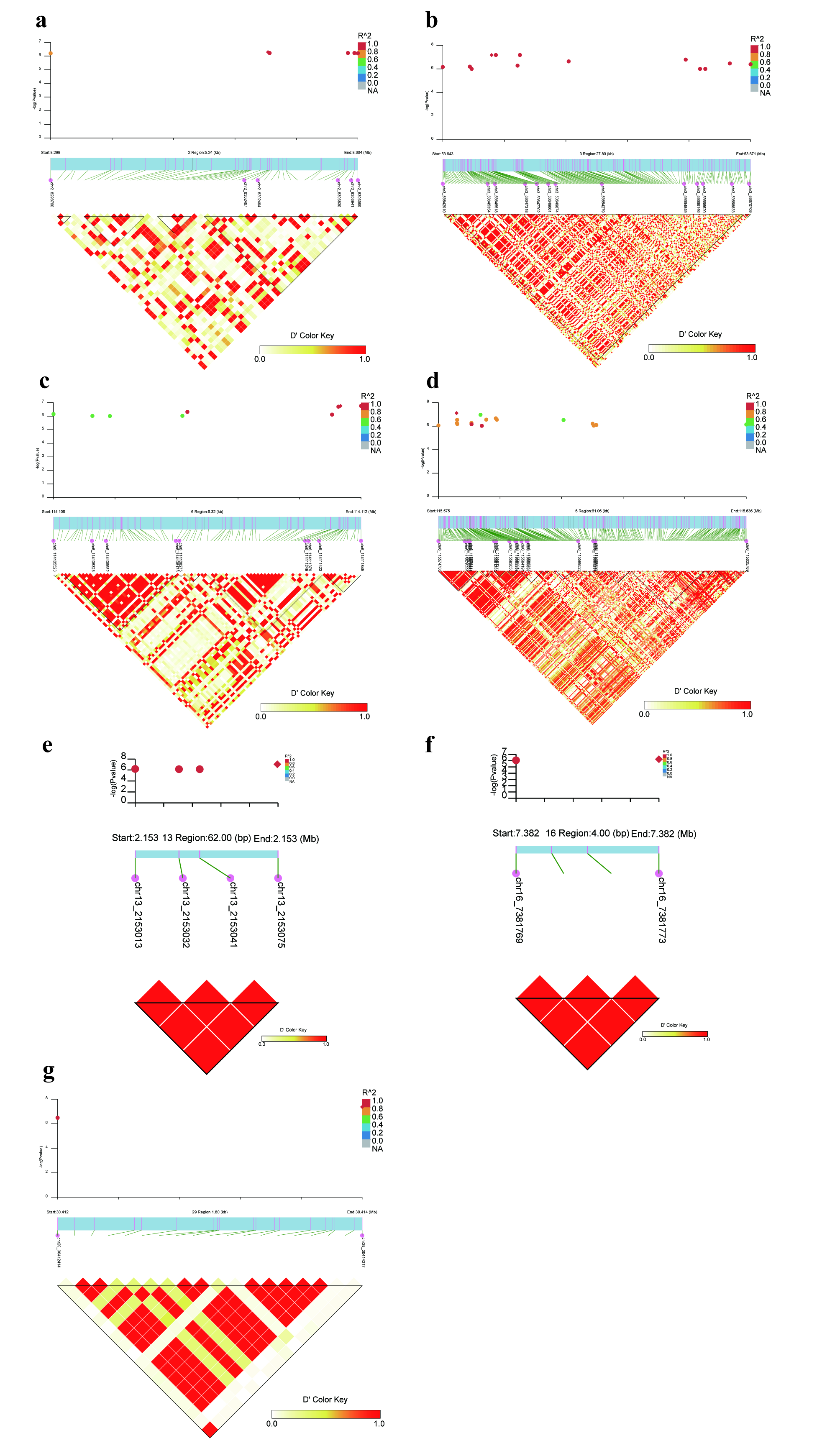


**Supplementary Figure 4.** Construction of haplotype blocks significantly associated with fleece length traits. **(a)** represents chr2_g.8302467T>C, chr2_g.8302494A>G, chr2_g.8303830C>T, chr2_g.8303941T>C, and chr2_g.8303999T>C are located in 1 Block; **(b)** represents chr3_g.53645354C>T, chr3_g.53645518G>T, chr3_g.53647318C>T, chr3_g.53647702T>C, chr3_g.53649661A>C, chr3_g.53649874C>T, chr3_g.53654279G>C, chr3_g.53664849A >G, chr3_g.53666146G>C, chr3_g.53666620T>A, and chr3_g.53668833C>T are located in 1 Block; **(c)** represents chr6_g.114106323C>T, chr6_g.114106682G>T, and chr6_ g.114108173C>T are located in 1 Block; chr6_g.114111249A>G, chr6_g.114111379G>A and chr6_g.114111423A>G are located in 1 Block; **(d)** represents chr6_g.115574706C>T , chr6_g.115578298C>G, chr6_g.115578368G>A, chr6_g.115578415A>C, and chr6_g.115578444G>A are located in 1 Block; chr6_g.115581252G>A and chr6_ g.115581272C>A are located in 1 Block; chr6_g.115583050G>T, chr6_g.115583302T>C and chr6_g.115583312G>A are located in 1 Block; chr6_g.115584191T>A , chr6_g.115586088G>C and chr6_g.115586283G>A are in 1 Block; chr6_g.115605297A>G, chr6_g.115605508T>C and chr6_g.115606026T>C are in 1 Block; **(e)** represents chr13_g.2153013A>G, chr13_g.2153032T>G, chr13_g.2153041T>C and chr13_g.2153075T>C are located in 1 Block; **(f)** represents chr16_g.7381769G>A and chr16_ g.7381773C>T are located in 1 Block; **(g)** represents chr29_g.30412414G>A and chr29_g.30414217T>C are located in 1 Block.
